# Sodium-bile acid co-transporter is crucial for survival of a carcinogenic liver fluke *Clonorchis sinensis* in the bile

**DOI:** 10.1371/journal.pntd.0008952

**Published:** 2020-12-07

**Authors:** Fuhong Dai, Won Gi Yoo, Yanyan Lu, Jin-Ho Song, Ji-Yun Lee, Youngro Byun, Jhang Ho Pak, Woon-Mok Sohn, Sung-Jong Hong

**Affiliations:** 1 Department of Medical Environmental Biology, Chung-Ang University College of Medicine, Seoul, Republic of Korea; 2 Department of Parasitology, School of Biology and Basic Medical Sciences, Medical College, Soochow University, Suzhou, Jiangsu, PR China; 3 Liubei Center for Disease Control and Prevention, Liuzhou, Guangxi, PR China; 4 Department of Pharmacology, Chung-Ang University College of Medicine, Seoul, Republic of Korea; 5 Research Institute of Pharmaceutical Sciences, College of Pharmacy, Seoul National University, Seoul, Republic of Korea; 6 Department of Convergence Medicine, University of Ulsan College of Medicine and Asan Institute for Life Sciences, Asan Medical Center, Seoul, Republic of Korea; 7 Department of Parasitology and Tropical Medicine, and Institute of Health Sciences, Gyeongsang National University College of Medicine, Jinju, Republic of Korea; National University of Ireland Galway, IRELAND

## Abstract

The liver fluke *Clonorchis sinensis* inhabits the bile ducts, where bile concentration disparities across the fluke cell membrane can cause bile intoxication. Sodium-bile acid co-transporter (SBAT) plays a crucial role in bile acid recycling. The process by which SBAT imports bile acids is electrically coupled to sodium ion co-transportation. Here, we report that the SBAT of *C*. *sinensis* (CsSBAT) is involved in bile acid transportation. CsSBAT cDNA encoded a putative polypeptide of 546 amino acid residues. Furthermore, CsSBAT consisted of ten putative transmembrane domains, and its 3D structure was predicted to form panel and core domains. The CsSBAT had one bile acid- and three Na^+^-binding sites, enabling coordination of a symport process. CsSBAT was mainly localized in the mesenchymal tissue throughout the fluke body and sparsely localized in the basement of the tegument, intestinal epithelium, and excretory bladder wall. Bile acid permeated into the adult flukes in a short time and remained at a low concentration level. Bile acid accumulated inside the mesenchymal tissue when CsSBAT was inhibited using polyacrylic acid–tetradeoxycholic acid conjugate. The accumulated bile acid deteriorated the *C*. *sinensis* adults leading to death. *CsSBAT* silencing shortened the lifespan of the fluke when it was placed into bile. Taken together, we propose that CsSBAT transports bile acids in the mesenchymal tissue and coordinate with outward transporters to maintain bile acid homeostasis of *C*. *sinensis* adults, contributing to *C*. *sinensis* survival in the bile environment.

## Author summary

Clonorchiasis is a neglected tropical disease caused by infection with the liver fluke *Clonorchis sinensis*. *C*. *sinensis* is a biological carcinogen causing cholangiocarcinoma in humans. Juvenile worms inhabit and grow to adults in the bile ducts. Bile acids in the bile are double-edged molecules; they promote metabolism, but differences in their concentration across the cell membrane could lead to bile intoxication. The sodium-bile acid co-transporter of *C*. *sinensis* (CsSBAT) is indispensable for maintaining its normal physiology and bile detoxification in the bile duct. However, information related to the molecular and biological characteristics of the SBAT of liver flukes is not available. Here, we cloned CsSBAT for the first time in trematodes and characterized its tertiary structure and physiological functions. The sequential and structural properties of CsSBAT were similar to the apical sodium-bile acid co-transporter found in mammalian intestines. CsSBAT shared a mesenchymal tissue distribution with Na^+^-taurocholate co-transporting polypeptide in the hepatocytes adjacent to the bile ducts. Bile acids accumulated in *C*. *sinensis* adults when CsSBAT was inhibited, causing their death. This information might promote further studies on the physiological functions of SBAT and other trematode bile transporters and open new avenues toward developing novel anthelminthic drugs.

## Introduction

Human clonorchiasis is caused by the liver fluke *Clonorchis sinensis*, an endemic trematode parasite. It is one of the major neglected tropical diseases and remains a public health problem especially in Asian countries. People are infected with *C*. *sinensis* by ingesting raw or undercooked freshwater fish infected with metacercariae [1]. Over 35 million people have been infected by *C*. *sinensis* in Asian countries, including Korea, China, and Vietnam [2–4]. The complications associated with clonorchiasis are dependent on the intensity and duration of infection. The World Health Organization has recognized *C*. *sinensis* as a biological carcinogen leading to human cholangiocarcinoma [5].

*Clonorchis sinensis* live in bile, which presents an extreme environment for their survival [6]. Bile acids in the bile are physio-chemically double-edged molecules. They are involved in various biological processes such as fatty acid uptake and lipid and cholesterol discharge [7]. In addition, they act as signaling molecules on bile acid-activated receptors including nuclear receptor and G protein-coupled receptor that are involved in innate immunity and neurodegenerative disorders [8,9]. However, imbalance in the bile acid influx and efflux can cause bile intoxication [10]. In humans, bile acid transporters such as apical sodium-dependent bile acid transporter (ASBT), Na^+^-taurocholate co-transporting polypeptide (NTCP), multidrug resistance protein (MRP), bile salt export pump (BSEP), and organic solute transporter (OST) are essential elements for maintaining the bile acid pool through enterohepatic bile circulation [11,12].

The sodium-bile acid co-transporters (SBATs) are subfamily members of the solute carrier family 10 (SLC10), whose bile acid uptake is coupled with sodium ion co-transport [13]. In higher animals, SBATs play a crucial role in the enterohepatic bile salt circulation. Bile acids are taken up by NTCP at the sinusoidal membrane in hepatocytes and released into the bile duct by BSEP and MRP [13]. A small fraction of bile acids is absorbed by biliary epithelium cholangiocytes before being sent back to the hepatocytes. This transcellular transport of bile acids across the biliary epithelium is mediated by ASBT on the apex and OSTα/β on the basolateral membrane [14]. In the ileum, most bile acids are reabsorbed through ASBT and pumped out into the portal blood by OSTα/β and MRP3 [11,12]. In the kidney, ASBT reabsorbs bile acids on the proximal tubules and returns them to the liver [15].

The structural and functional elements of human ASBT (HsASBT) have been identified [13], but its crystal structure has not yet been elucidated. Among the transmembrane (TM) domains, both TM3 and TM4 can interact with bile acids on the extracellular half of the membrane, while TM6 and TM7 can interact with them on the intracellular half of the membrane [16–19]. In contrast, TM2 and TM5 take part in sodium ion translocation and stabilization [20,21]. Recently, the tertiary structure of ASBT homologs in *Neisseria meningitidis* [22] and *Yersinia frederiksenii* [23] have been modeled to have inward facing and outward facing conformations. These findings provided a clue to understanding the mechanism behind the sodium ion and bile acid translocation across the cell membrane. Structural features of NTCP were analyzed using bacterial ASBT as a template [24,25].

Although functional studies on bile transporters of the platyhelminths are rare, a large amount of genetic information on the bile transporters can be retrieved from the GenBank database, including sodium-bile acid symporter family protein (Acc. ID: OON14614.1) in *Opisthorchis viverrini*, SLC10 member 6 (Acc. ID: THD25133.1) in *Fasciola hepatica*, sodium/bile transporter member 3/5 (Acc. ID: KAA3675191.1) in *Paragonimus westermani*, and sodium-bile acid co-transporter (Acc. ID: CDS43508.1) in *Echinococcus multilocularis*. Thus, studies are required to fill the large gap between functional studies and the increasing number of sequence resources.

Recently, two bile transporters were reported from *C*. *sinensis*: CsMRP4 and CsOST. CsMRP4 is the adenosine triphosphate (ATP)-dependent efflux transporter of various bile acids, belonging to the ATP-binding cassette transporter family [26,27]. CsOST was reported as an exporter disseminating bile acids by revealing evolutionarily conserved structural similarity [28]. The bile transporters show a tissue-specific distribution, implying that they orchestrate to maintain bile acid homeostasis [27–29]. Along with ASBT, OSTα/β plays an important role in regulating bile acids [11].

This study was performed to identify and characterize SBAT from a liver fluke *C*. *sinensis*. Initially, we obtained a CsSBAT 3D model using homology modeling and refinement. Then, we unraveled the CsSBAT function to understand how it affects *C*. *sinensis* survival in the bile.

## Materials and methods

### Ethics statement

The experimental rabbits (New Zealand white, male, 2–3 kg) and BALB/c mice (male, 7-week-old) were handled in an accredited Chung-Ang University animal facility (Accredited Unit, Korea FDA; Unit Number 36) in accordance with the Association for Assessment and Accreditation of Laboratory Animal Care International Animal Care policies. Approval for animal experiments was obtained from the Institutional Review Board of Chung-Ang University animal facility (Approval Number CAU-2014-00024 and CAU-2015-00005).

### CsSBAT cDNA and polypeptide sequences

In the process of searching for bile acid transporters in the *C*. *sinensis* transcriptome database [30], a cDNA clone (CSA06873) homologous to SBAT C-terminal half was found. Its glycerol stock was shared from the *C*. *sinensis* EST (expressed sequence tag) library bank in Korea Centers for Disease Control and Prevention [31]. A plasmid containing the EST was sequenced and confirmed to encode C-terminal region of the SBAT. We searched for a full-length cDNA clone homologous to the EST clone (CSA06873), and found one hypothetical CsSBAT mRNA and genomic sequence (Acc. ID: GAA57409) in the NCBI GenBank database. To obtain a full-length cDNA of CsSBAT, 5′-RACE was performed. Primers were designed referring to the hypothetical CsSBAT mRNA sequence. A forward primer including the first methionine was designed as 5′-ATGTTGCTTCGGTGGGCTTGGCT-3′. A reverse primer was designed as 5′-TCACAGCCTACTATCATGGAAGTGT-3′ on 5′-end of the partial cDNA clone. *C*. *sinensis* total cDNA was used as template for 5′-RACE PCR. Total RNA was extracted from adult *C*. *sinensis* using TRIzol reagent (Ambion, CA, USA). Trace genomic DNA contamination was removed using the DNA-free kit (Ambion, CA, USA). RNA quality was assessed using agarose-formaldehyde gel electrophoresis. RACE-ready first-strand cDNA was synthesized using the SMARTScribe reverse transcriptase kit (Clontech, Mountain View, CA, USA). Using the cDNA as template, the anterior half of CsSBAT cDNA was amplified using PCR and purified using QIAquick PCR purification kit (QIAGEN, Seoul, Korea). The purified PCR product was subcloned into the pCR2.1-TOPO vector using the TOPO TA cloning kit (Invitrogen, Carlsbad, CA, USA). A white colony was picked and cultured overnight. Plasmid DNA was extracted using a plasmid miniprep kit (QIAGEN, Hilden, Germany) and further sequenced. After the full-length CsSBAT cDNA was obtained, it was translated to the amino acid sequence to obtain the open-reading frame.

### Sequential and phylogenetic analyses

A disordered region was predicted on the putative CsSBAT polypeptide sequence using DISOPRED3 [32]. Homologous proteins/polypeptides were retrieved using BLASTP search [33], and the canonical SLC10A family member proteins were retrieved from UniProtKB/Swiss-Prot database [34]. All sequences were multiple aligned with the parameters of L-INS-i method and BLOSUM62 scoring matrix using MAFFT v7.299 [35], and displayed using Jalview [36]. An evolutionary history of homologous proteins and SLC10A family proteins was inferred using the maximum-likelihood method. Clustering probability with taxa was calculated using a bootstrap test with 1,000 replicates. Evolutionary history was analyzed using MEGA7 [37].

### Tertiary structure modeling and refinement

The three-dimensional (3D) structure of CsSBAT was generated using homology modeling with Swiss-Model [38]. The 3D model was refined in two steps. Low free-energy conformations of the 3D model were refined using full-atomic simulations with FG-MD [39]. The backbone and side chains of the 3D model were refined using GalaxyRefine [40] with “both mild and aggressive relaxation” method based on repeated perturbation and overall conformational relaxation with short molecular dynamics simulations.

### Quality validation and structural characterization of the 3D model

The structural quality of 3D models was evaluated using PROCHECK [41], ProSA [42], and ERRAT [43]. The residue-by-residue stereochemical quality of 3D models was validated using the Ramachandran plot in PROCHECK. The overall quality score was verified by calculating the model’s atomic coordinates using ProSA with a Z-score of crystallographic structures in the Protein Data Bank (PDB) database [44]. Statistics of non-bonded atom–atom interactions were analyzed using a database of high-resolution crystallographic structures with ERRAT [43].

The secondary structural elements and topology of CsSBAT were analyzed using ProFunc [45]. The secondary structure of CsSBAT was determined based on that of *Neisseria meningitidis* ASBT (GenBank Acc. ID: WP_002244055.1). Several aligned regions of CsSBAT with structurally similar proteins were searched using iPBA [46]. Evolutionary conservation was calculated using ConSurf [47], and conservation values were mapped onto 3D structures. The structure was visualized using UCSF Chimera [48].

### Metacercariae and adult worms of *C*. *sinensis*

*Pseudorasbora parva* (Jinju, Korea), the second intermediate *C*. *sinensis* host, were ground and digested in artificial gastric juice [8 g of pepsin 1:10,000 (MP Biochemicals Co., Solon, OH, USA) and 8 mL of concentrated HCl in 1 L of water] at 37°C for 2 h [49]. Rough matter was removed from the digested content by filtering through a 212 μm mesh sieve. *C*. *sinensis* metacercariae were harvested using sieves of 106 and 53 μm mesh, washed thoroughly several times with 0.85% saline, and collected under a dissecting microscope. Rabbits (New Zealand White, male, 2–3 kg; Koatech, Seoul, Korea) were each fed with 200 *C*. *sinensis* metacercariae, and they were fed again after one week. Several months later, *C*. *sinensis* adult flukes were recovered from the bile duct of these rabbits and stored at −20°C until use.

### Reverse-transcription quantitative PCR (RT-qPCR) and relative quantification of CsSBAT mRNA

The frozen *C*. *sinensis* adults and metacercariae were pulverized in liquid nitrogen. Total RNA was extracted using TRIzol reagent (Ambion, CA, USA) and treated with the DNA-free kit (Ambion, CA, USA) to remove traces of genomic DNA according to the manufacturer’s instructions. RNA quality indicated by OD_260/280_ ratio was assessed using a spectrophotometer (Ultrospec 3000, Pharmacia Biotech, Amersham, UK) and confirmed to be above 1.9. First-strand cDNA was synthesized using total RNA, oligo-d(T) primer, and Power cDNA Synthesis kit (iNtRON Biotechnology, Gyeonggi-do, Korea). Developmental transcription of *CsSBAT* in the adults and metacercariae was determined using RT-qPCR. Primers were designed using Oligo-primer analysis software v6.71 (Molecular Biology Insights, Cascade, WA, USA). A set of primers to prime the 374 bp fragment of CsSBAT cDNA were designed as follows: CsSBAT-F, 5′-GTCCGTGAGGCGAGAAACTTG-3′, and CsSBAT-R, 5′-AGCAGACCAAAACCAAACTCC-3′. The β-actin, calcyphosine, and phosphoglycerate kinase genes were employed as reference standards to compare between developmental stages [50], and we used the same primer pairs of these genes as those used in the reference [50] for our RT-qPCR. An equal amount (50 ng) of total cDNA from the adults or metacercariae and primers were mixed with 1 μL of 10× master mix (FastStart SYBR Green I Kit, Roche, Mannheim, Germany) to achieve a final volume of 10 μL. Thermal cycling was performed on a LightCycler 2.0 (Roche, Mannheim, Germany) using the following cycling conditions: 95°C for 15 min followed by 45 cycles of 95°C for 10 s, 60°C for 10 s, and 72°C for 30 s. To plot a melting curve, PCR products were heated to 95°C for 10 s, cooled to 65°C, and heated again to 95°C with an increment of 0.1°C/s. Data were analyzed using the LightCycler program. The relative transcription/expression level was calculated using the 2^−ΔΔCt^ equation [51].

### CsSBAT chimeric protein for antibody production

A chimeric protein was designed to produce highly immunogenic and soluble recombinant antigen. The CsSBAT polypeptide sequence was analyzed using the BepiPred linear epitope prediction program (http://tools.immuneepitope.org/tools/bcell/iedb_input). Hydrophilicity was estimated using ProtScale (http://web.expasy.org/cgi-bin/protscale/protscale.pl). For the chimeric protein production, two regions simultaneously showing high epitope probability and high hydrophilicity were selected at N- and C-termini of CsSBAT (S1 Fig). Two cDNA fragments corresponding to the antigenic regions were PCR-amplified on the CsSBAT cDNA using appropriate primer sets. Endonuclease enzyme restriction sequence was added to each 5′-end of the primers to favor subcloning the PCR-amplified cDNA fragment into a plasmid expression vector. An oligo-peptide, GPGPG spacer, was incorporated between the two antigenic epitopes (S1 and S2 Figs) to enhance protein stability and antibody accessibility [52,53].

One primer set of the N-terminal epitope including the GPGPG spacer was designed as CsSBAT-cp1-*Bam*HI-F: 5′-GGGGATCCCAAGCGACCGTAGCCATACCA-3′ and CsSBAT-cp1-GPGPG-*Eco*RI-R: 5′-TGTGAATTCACCCGGGCCCGGACCGTGGCGCGATGGAAGATT-3′. Another primer set of the C-terminal epitope was CsSBAT-cp2-*Eco*RI-F: 5′-TATGAATTCCTGTTGGGAGTCACGATCAA-3′ and CsSBAT-cp2-*Hin*dIII-R: 5′-ATAAGCTTCGCCCTACTATCATGAAAGTGT-3′. Each target region of the CsSBAT cDNA was PCR-amplified using the corresponding primer set. The amplicons were double-digested using the respective restriction endonucleases. The digested CsSBAT-cp2 cDNA fragment was first ligated into a plasmid expression vector pRSET A. Next, the restriction endonuclease-double digested CsSBAT-cp1-GPGPG cDNA fragment was directionally ligated into the newly constructed plasmid CsSBAT-cp2-pRSET A, in which the chimeric protein was produced as a histidine-tagged chimeric fusion protein (S2 Fig). This chimeric expression construct, pRSET-CsSBAT-cp1-GPGPG-CsSBAT-cp2-His, was transformed into *E*. *coli* DH5α competent cells (Takara Korea Biomedical Inc., Seoul, Korea) and selected on LB/ampicillin plates. Fidelity of the chimeric expression construct was verified by sequencing the recombinant plasmid DNA (Macrogen, Seoul, Korea).

The plasmid DNA of the chimeric CsSBAT construct was transformed into *E*. *coli* BL21[DE3]pLysS (Novagen, San Diego, CA) by heat shock at 42°C for 30 s, and spread on LB/ampicillin (50 μg/mL) plates. A single colony was inoculated into LB liquid medium and cultured overnight at 37°C until OD_600_ value reached 0.6. The chimeric protein production was induced by adding isopropyl-β-d-thiogalactopyranoside (Takara, Shiga, Japan) at a final concentration of 0.1 mM, and the bacteria were further cultured for 3–5 h. The bacterial cells were harvested by centrifugation and sonicated in native lysis buffer (1× PBS, pH 7.4, 1% Triton X-100) on ice. After centrifugation at 13,000 rpm for 10 min at 4°C, lysate supernatant was loaded onto a Ni-NTA column (QIAGEN, Hilden, Germany). The CsSBAT chimeric protein was eluted with 1× PBS containing 200 mM imidazole, dialyzed against 1× PBS, and resolved on a 12.5% SDS-PAGE gel.

### Anti-CsSBAT chimeric protein antibody production

The eluted CsSBAT-chimeric protein fraction was separated using SDS-PAGE and the target band was cut out as a slice. This gel slice holds the protein of much higher purity than the eluted fractions. The gel slice was equilibrated three times in sterile 1× PBS for 30 min and ground with a tissue grinder. The gel homogenate (200 μL) was injected into the abdominal cavity of BALB/c mouse (male, 7-week-old; Orient Bio Inc., Seongnam, Korea). After two weeks, the same amount of homogenate was injected into the other side of the abdominal cavity of the mouse. A booster (50 μL of the eluted antigen) was injected into the mouse tail vein two weeks later. Three days after the booster injection, one or two drops of mouse blood were collected from the tail vein and tested for specific antibody production against the CsSBAT chimeric protein using western blot.

Mice producing specific antibody were chosen, and blood was sampled from the orbital basin. Immune sera were collected after clotting and centrifugation, and stored at −20°C until use.

To detect native CsSBAT in the soluble extract, *C*. *sinensis* adults were homogenized in two volumes of 1× PBS containing 1% Triton X-100 and 1× CompleteMini using a Tissue Tearor (Model 987–370, Biospes Products, Bartlesville, OK, USA) on ice. The homogenate was kept at 4°C overnight and centrifuged at 700 × *g* for 5 min at 4°C. Supernatant was saved as *C*. *sinensis* soluble extract. Protein concentration of the soluble extract was determined by spectrophotometry at OD_595_.

The soluble extract of *C*. *sinensis* adults was run on 12.5% SDS-PAGE gel and transferred onto nitrocellulose membrane (Amersham Hybond-ECL membrane, GE Healthcare Bio-Sciences, Uppsala, Sweden). The CsSBAT chimeric protein was run as a positive control. The CsSBAT-chimeric and native CsSBAT proteins on the membrane were detected using the mouse immune serum at a 1:200 dilution and a secondary antibody, goat-anti-mouse-POD IgG (Sigma, Steinheim, Germany) at a 1:3,000 dilution. Immuno-reactive proteins were detected using an enhanced chemiluminescence (ECL) kit (Bio Sesang, Seoul, Korea) and visualized using ImageQuant LAS 4000 (GE Healthcare Bio-Sciences).

### Immunohistochemical staining

The rabbit liver infected with *C*. *sinensis* adults was resected, sliced into small pieces, and fixed in 10% buffered formalin (pH 7.0) for 4–8 h. The fixed liver slices were paraffin embedded, sectioned, and processed for further experiments. Mouse anti-CsSBAT-chimera immune serum at 1:200 dilution in antibody dilution solution (Life Science Division, WA, USA) was used as a primary antibody. After washing, the sections were incubated with horseradish peroxidase-labeled polymer anti-mouse IgG antibody (diluted at 1:400) (EnVision+ System, Dako Cytomation, Glostrup, Denmark) at room temperature for 1 h. Color was developed using 3-amino-9-ethylcarbazole as substrate. The sections were counterstained with hematoxylin.

Immunofluorescence staining was performed for further validation. Mouse anti-CsSBAT-chimera immune serum at 1:200 dilution was added to the liver sections and incubated at 4°C overnight. After washing in a dark room, the sections were incubated with Alexa Fluor 488-labeled goat anti-mouse IgG (Invitrogen, Rockford, IL, USA) and rinsed three times with 1× Tris-buffered saline (each for 10 min). The sections were mounted in anti-fade mounting reagent (Ultramount aqueous permanent mounting, Dako, CA, USA), and fluorescence signals were recorded under LSM 700 confocal microscope and ZEN 2011 (Carl Zeiss, Jena, Germany).

### CsSBAT inhibition and survival assay on *C*. *sinensis* adults

To allow to regurgitate the intestinal contents, the *C*. *sinensis* adults were incubated in 1× Locke’s solution [54] at 37°C with 5% CO_2_ for 24 h. Then, ten active flukes were incubated in 2.5 mL 1× Locke’s solution/well on a 6-well plate. Assay groups each had three wells. Polyacrylic acid–tetradeoxycholic acid conjugate (PATD), an effective inhibitor of human ASBT [55], was employed as a CsSBAT inhibitor.

The *C*. *sinensis* adults in the experimental group were incubated in 1× Locke’s solution containing 0.075% bile (bovine; Sigma-Aldrich, St. Louis, MO, USA) for 5 min, and PATD was added to a final concentration of 100 μg/mL. Three control groups were set up as 1× Locke’s solution, 1× Locke’s solution with 100 μg/mL PATD, and 1× Locke’s solution with 0.075% bile. An antibiotic (Gibco Antibiotic-Antimycotic, Gibco/Invitrogen, CA, USA) was added to all incubation solutions as instructed on the product sheet. The solution in the well was replaced with fresh solution every 12 h.

The inhibitory effects of PATD on CsSBAT in the *C*. *sinensis* adults were assayed based on their survival and motility. Motility was arbitrarily scored from grade 1 to 5, from the most passive to the most active. The flukes showing no visible response to gentle stimuli using wooden applicator were considered dead [54,56]. The flukes were observed every 12 h, and the number of surviving *C*. *sinensis* and motility score were recorded for 8 days. The assay was performed in biological triplicate.

To trace movement of bile acids into the *C*. *sinensis* adults, a fluorescent bile acid derivative, *N*-(24-[7-(4-*N*,*N*-dimethylaminosulfonyl-2,1,3-benzoxadiazole)]amino-3α,7α,12α-trihydroxy-27-nor-5β-cholestan-26-oyl)-2′-aminoethanesulfonate (tauro-nor-THCA-24-DBD; GenoMembrane, Yokohama, Japan), was employed. Active flukes were incubated in 1.5 mL 1× Locke’s solution with five flukes/well on a 24-well plate. As preliminary experiments, tauro-nor-THCA-24-DBD was added at 1, 5, 25, 125, 250, and 500 μM and incubated for 10 min. Time-dependent movement of fluorescent bile acid in the *C*. *sinensis* adults was measured at 2.5, 5, and 10 min.

For inhibitory assays, experimental groups were set up with 25 μM tauro-nor-THCA-24-DBD and 400 μg/mL PATD and incubated for 5 min. Control groups were incubated in 1× Locke’s solution containing 25 μM tauro-nor-THCA-24-DBD or 400 μg/mL PATD. The mock control group was incubated in 1× Locke’s solution. Then, all the flukes were fixed in 0.5% paraformaldehyde and gently rinsed three times with 1× Locke’s solution containing 25 μM taurocholic acid. The flukes were mounted in Gel Mount (Biomeda, CA, USA). In a darkroom, one antero-lateral part of the *C*. *sinensis* adults, in which organs less presented, were observed under a confocal microscope (LSM 700, Carl Zeiss, Heidelberg, Germany). Fluorescence was measured at an excitation wavelength of 488 nm and at an emission wavelength of 570 nm. Fluorescence intensity (FI) was quantified using ZEN lite 2011 (Blue edition, version 1.0.0.0). Background FI of the negative control was deducted from that of all groups. All of the tracing and inhibition experiments were performed in triplicate.

### RNA interference (RNAi)

N- and C-terminal sequences of CsSBAT cDNA were scrutinized to produce CsSBAT-specific double-strand RNA (dsRNA). Two regions, 213 bp and 168 bp on each terminus, were selected as templates for dsRNA synthesis using T7 polymerase-driven transcription (S3A Fig). PCR primers, forward and reverse, were extended at the 5′-end with T7-polymerase promoter sequence (5′-TAATACGACTCACTATAGGGAGA-3′) for the following dsRNA synthesis. PCR primers for dsRNA1 were T7-dsRNA1-forward 5′-TAATACGACTCACTATAGGGAGACAAGCGACCGTAGCCATA-3′ and T7-dsRNA1-reverse 5′-TAATACGACTCACTATAGGGAGAGTCAGTTAGGCGCAGATCA-3′. For dsRNA2, the primers were T7-dsRNA2-forward 5′-TAATACGACTCACTATAGGGAGACTGTTGGGAGTCACGATCAA -3′ and T7-dsRNA2-reverse 5′-TAATACGACTCACTATAGGGAGAGTGTTTCGTACCATTCGGTT-3′. Amplicons of the two PCRs were purified using the QIAquick gel extraction kit (QIAGEN, Hilden, Germany) and used for dsRNA synthesis with the MEGAshortscript kit (Invitrogen, Vilnius, Lithuania). CsSBAT-single-stranded RNA 1 (CsSBAT-ssRNA1) and 2 (CsSBAT-ssRNA2) were synthesized according to the manufacturer’s instructions. To formulate dsRNA, a transcription reaction mix containing sense- and antisense-ssRNA of each template was incubated promptly in 75°C water bath for 5 min and maintained at room temperature for 30 min. dsRNA was formed by annealing the sense- and antisense-ssRNAs during the cooling period. To purify dsRNA and degrade residual DNAs and single-strand RNAs, the annealing products were treated with TURBO DNase (Invitrogen, Vilnius, Lithuania) and RNase I_f_ (New England Biolabs, Ipswich, MA, USA) at 37°C for 15 min [57]. After phenol/chloroform extraction and ethanol precipitation, the dsRNA pellet was dissolved in DEPC-treated H_2_O (Biosesang, Seongnam, Gyeonggi-do, Korea) and stored at −20°C. Quality and quantity/concentration of the dsRNA1 and dsRNA2 were evaluated using the NanoDrop 1000 spectrophotometer (Thermo Scientific, Wilmington, DE, USA). The concentration of the 1:3 diluted dsRNAs was over 2,700 ng/μL. dsRNAs with 260/280 and 260/230 ratios higher than 1.8 were employed for downstream RNAi experiments.

Our RNAi experiment was performed with reference to HsASBT and HsNTCP because CsSBAT was highly homologous to them. In the human-derived cell cultures, HsNTCP and HsASBT mRNA and protein revealed half-lives of 6–24 h [58,59]. In RNAi-mediated gene silencing experiments, the liver flukes were soaked in the media containing dsRNAs. The dsRNAs reduced the target gene transcripts significantly at low concentration and in short soaking time. With one initial soaking, the dsRNAs silenced the target genes effectively for several days [57,60].

The *C*. *sinensis* adults, 20 flukes/well, were soaked in 3 mL of 1× Locke’s solution containing 100 ng/μL dsRNA1 or dsRNA2 in a 6-well plate for 6 h at 37°C. A control group was set with dsRNA-untreated *C*. *sinensis* adults in 1× Locke’s solution. Total RNA was extracted from each group of the *C*. *sinensis* adults, and the CsSBAT mRNA level was measured using RT-qPCR with the same thermal cycle and primers as described above.

After 6 h-soaking, the dsRNA-treated *C*. *sinensis* adults were subjected to CsSBAT RNA silencing assay *in vitro* for survival and motility in 0.075% bile in 1× Locke’s solution. As control groups, the ds-RNA-untreated flukes were incubated in 0.075% bile in 1× Locke’s solution or in 1× Locke’s solution, with the media replaced every day. The survival and motility of *C*. *sinensis* adults in the respective media were observed and recorded at 37°C every 12 h for 9 days as described above in the PATD inhibitory assay. The RNAi assay was performed in triplicate.

### Statistical analysis

Data were analyzed using Student’s *t*-test, and values of *p* < 0.05 were considered statistically significant. All values represent mean ± standard error. The mean was calculated from more than three independent assays.

## Results

### CsSBAT polypeptide and functional conservation

After sequencing, an expressed sequence tag clone CSA06873 of 888 bp was found to encode a polypeptide homologous to the C-terminal half of ASBTs, containing a 3′-untranslated region of 283 bp including a poly(A)-tail. Using the total cDNA obtained from *C*. *sinensis* as a template, the 5′-half of the cDNA was amplified using PCR. Translation of the full-length cDNA (1,924 nucleotides) revealed a putative polypeptide of 546 amino acids including the first methionine (S4 Fig). We searched for CsSBAT homologs in GenBank, finding only one hypothetical mRNA sequence (Acc. ID: GAA57409.1) in the *C*. *sinensis* draft genome information [61]. A deduced polypeptide of the hypothetical SBAT mRNA had three amino acid variations (M48V, V141A, and H179Y), differing from our CsSBAT (S4 Fig). These three variations were supposed not to interact with sodium ions and bile acids nor to affect the structural topology. In this point, GAA57409.1 was considered to be a *CsSBAT* transcript with three nucleotide polymorphisms. The full-length CsSBAT cDNA sequence was deposited to GenBank under the accession number KX756671 (S4 Fig).

**Fig 1:**
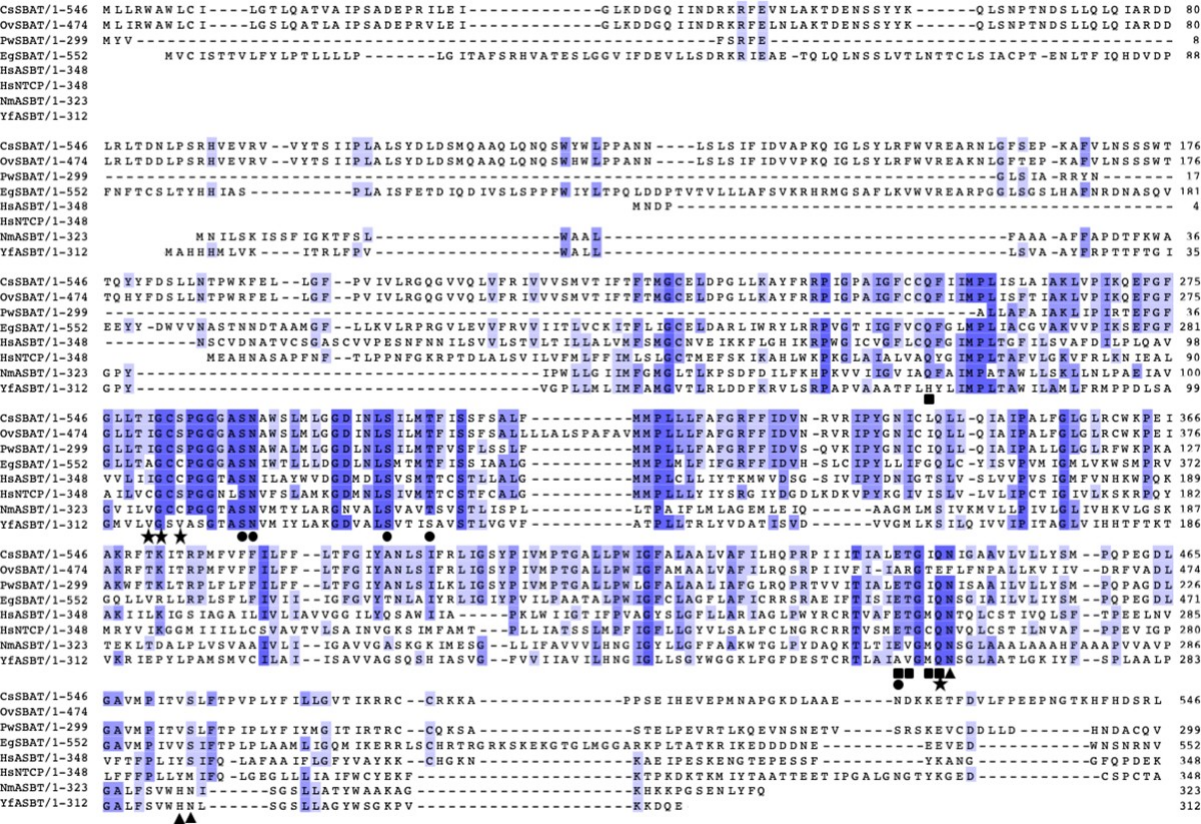
Multiple polypeptide alignment of CsSBAT and bile acid transporters from other species. Sequences were aligned and calculated using MAFFT v7.299 [35] with manual adjustment in Jalview [36]. Residues over 50% conserved among the transporters are shaded in light blue and 100% conserved residues are highlighted in dark blue. Residues that interact with sodium ions are labeled: Na1 site (●), Na2 site (■), and putative Na3 site (★). Residues necessary to coordinate with taurocholate in CsSBAT are also indicated (▲). Bile acid transporters are *Opisthorchis viverrini* SBAT (OvSBAT; A0A074Z5N1), *Paragonimus westermani* SBAT (PwSBAT; A0A5J4NIG5), *Echinococcus granulosus* SBAT (EgSBAT; W6UF08), human ASBT (HsASBT; Q12908), human NTCP (HsNTCP; Q14973), *Neisseria meningitidis* ASBT (NmASBT; Q9K0A9), and *Yersinia frederiksenii* ASBT (YfASBT; C4ST46).

The putative polypeptide sequence was compared to ASBT and NTCP sequences retrieved from the UniProtKB/Swiss-Prot database [62], and it showed a 27.1% similarity with HsASBT, 25.5% with HsNTCP, 21.5% with YfASBT, and 20.2% with NmASBT (Fig 1). All of the platyhelminth SBATs were hypotheticals deduced from the putative cDNAs edited with the draft genomic sequences in GenBank. The CsSBAT revealed conservation of the binding sites coordinating sodium ions and taurocholate with the NmASBT polypeptides. In the sodium ion binding sites, Glu_441_ was shared both with Na1 and Na2 sites, and Gln_445_ was shared with both Na2 and Na3 sites. Based on these characteristic features, this full-length cDNA was identified to encode CsSBAT.

A phylogenetic tree was constructed with CsSBAT homologs and canonical members of the SLC10A family (S5 Fig). The bile transporters of trematode clade including CsSBAT were distinct from those of cestode clade, although there were no well-characterized bile transporters in the platyhelminthic parasites. Vertebrate SLC10A members were clearly grouped together along with their subfamilies, while the invertebrate ASBTs like CsSBAT, NmASBT, and YfASBT were not grouped with any of the subfamily members (S5 Fig). Although CsSBAT elements were highly conserved, its N- and C-terminal regions differed from the others (S5 Fig), indicating that CsSBAT branched from the SLC10A subfamily members.

### 3D model of CsSBAT

Both N- and C-terminal regions of CsSBAT were predicted to be disordered (S6 Fig). These disordered regions can cause long simulation times and error-prone structural clustering [63]. Therefore, these regions were excluded from homology modeling. The functional region (residues 185–492 including all binding residues) of CsSBAT matched well with the reliable high-resolution crystallographic NmASBT (PDB ID: 3zuy_A) [22] and YfASBT structures (PDB ID: 4n7x_A) [23]. An initial 3D model of CsSBAT built using Swiss-Model [38] showed over 91.0% residues in the most favored region of a Ramachandran plot [64]. The model was subsequently improved by refinement. The final model was qualified by 92.5% residues in the favorable region and 6.3% residues in the allowed region in Ramachandran plot (S7A Fig). The final model’s ProSA Z-score was −3.73, showing that this was a strong, valid model (S7B Fig). The overall quality score from ERRAT was 97.7%, showing that the final model was a reasonably good model (S7C Fig). The structural quality of the CsSBAT 3D model was evaluated to be good compared to the recently reported data from homology modeling-based structures [65,66].

### CsSBAT topology and pseudosymmetry

**Fig 2:**
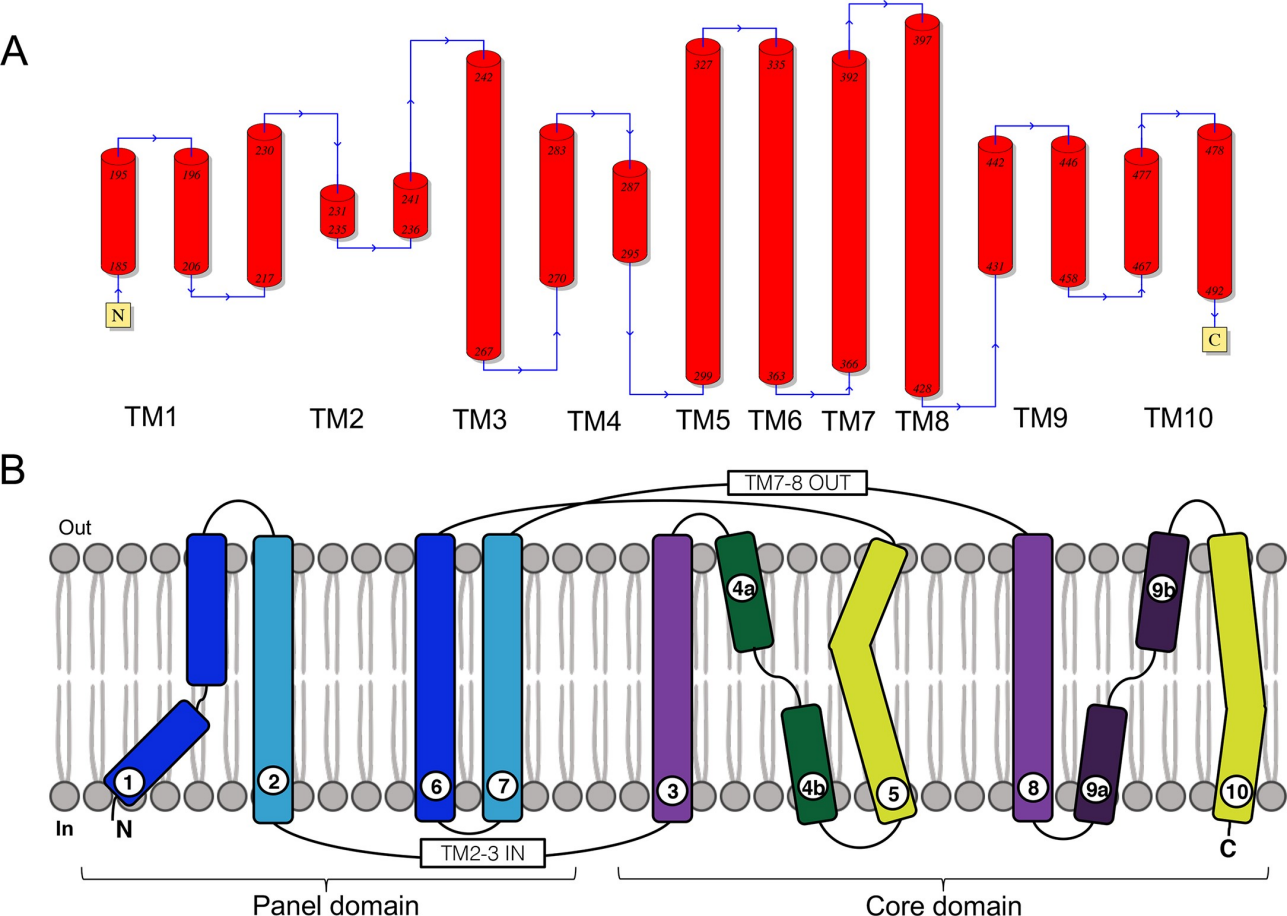
CsSBAT membrane topology and inverted structural repeats. (A) CsSBAT membrane topology was predicted based on the templates NmASBT (PDB ID: 3zuy_A) and ProFunc. Red barrels indicate α-helices. (B) N-terminal transmembrane domains (TMs) 1–2 and 3–5 were inversely repeated with C-terminal TMs 6–7 and 8–10.

**Fig 3:**
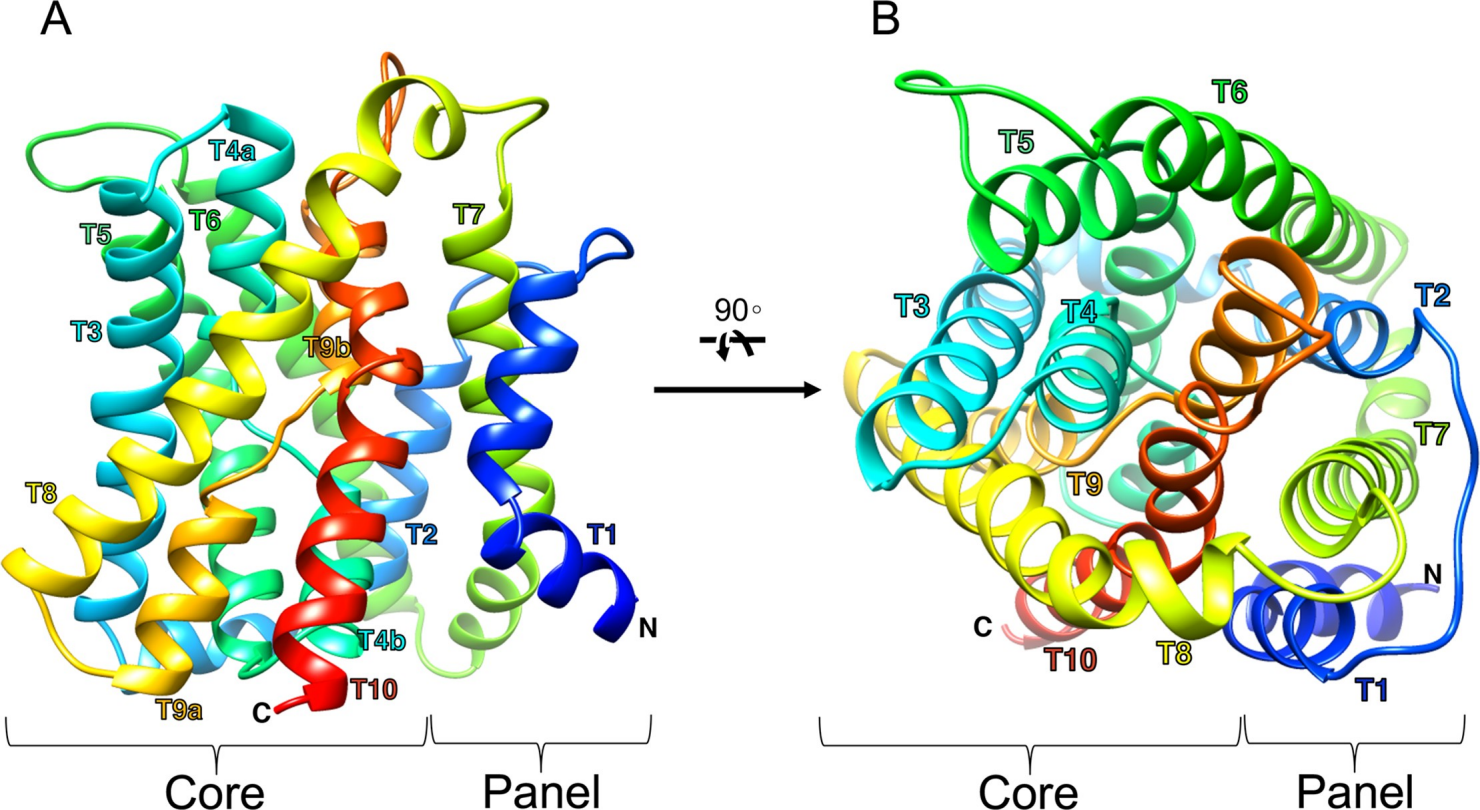
Ribbon representation of inward facing-CsSBAT 3D structure model. 3D structure was viewed from two perpendicular directions. (A) Plane of the membrane with the extracellular side up. (B) View from the extracellular side. Helices are colored from N-terminus in blue to C-terminus in red. Transmembrane domain is numbered as T#.

Similar to typical bacterial ASBTs [22,23], CsSBAT was predicted to consist of only α-helices contributing to ten transmembrane domains (TMs) connected with short loops. TMs 1, 2, 6, and 7 formed a panel domain, while TMs 3–5 and 8–10 formed a core domain (Figs 2 and 3). The first five TMs and the second five TMs were structurally similar to each other. They were symmetrically oriented in a plane of the cell membrane having internal two-fold pseudosymmetry (S8A–S8C Fig). The root-mean-square deviation (RMSD) of superposed anterior and posterior halves of CsSBAT was 2.79 Å. When the inverted repeats were superimposed, the panel and core domains had RMSD values of 2.68 Å and 2.58 Å, respectively (S8D and S8E Fig). TMs 4 and 9 were discontinuous and they crossed over into the core domain and the conserved region (S9 Fig).

### CsSBAT expression and distribution

CsSBAT mRNA was present both in the *C*. *sinensis* metacercariae and adults. The mRNA was 2.1 times more abundant in the metacercariae than in the adults (S10 Fig).

**Fig 4:**
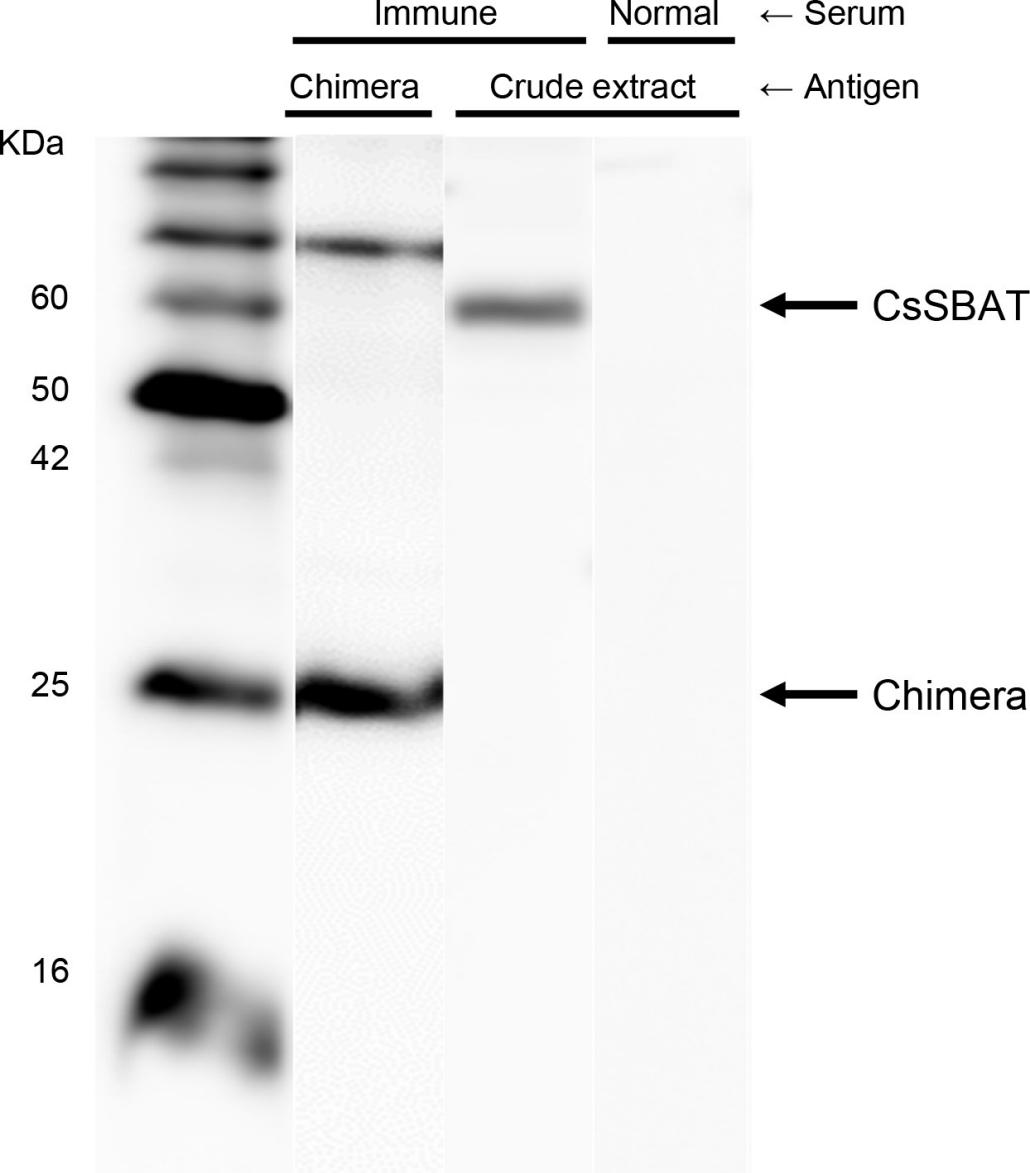
Reactivity of mouse anti-chimeric CsSBAT immune serum. The immune serum specifically detected native CsSBAT in an ECL immunoassay.

To produce chimeric antigenic protein, two cDNA fragments corresponding to putative B-cell epitopes and hydrophilic regions were amplified using PCR (S1, S2, S11A, and S11B Figs), ligated, and subcloned into an expression plasmid vector pRSET A. The plasmid expression construct was confirmed to contain the CsSBAT chimeric cDNA using restriction enzyme digestion (S11C Fig) and DNA sequencing. CsSBAT chimeric protein (24 kDa) was purified using Ni-NTA column (S12 Fig) and used for mouse immunization. A mouse immune serum specifically recognized native CsSBAT (60 kDa) in *C*. *sinensis* crude extract (Fig 4).

**Fig 5:**
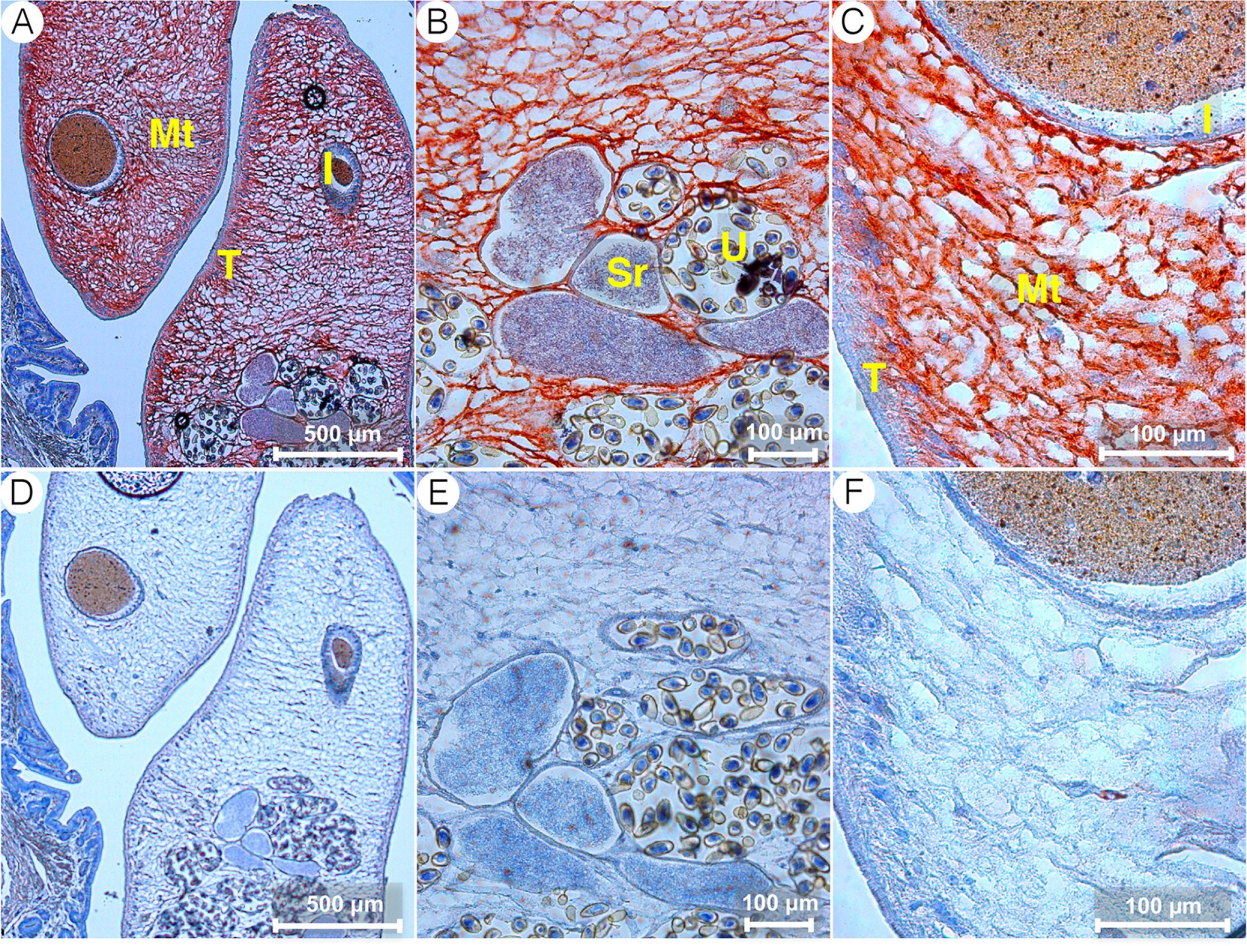
CsSBAT localization in *Clonorchis sinensis* adults using immunohistochemical staining. A–C, mouse anti-CsSBAT immune serum. D–F, normal mouse serum. I, intestine; Mt, mesenchymal tissue; Sr, seminal receptacle; T, tegument; U, uterus.

**Fig 6:**
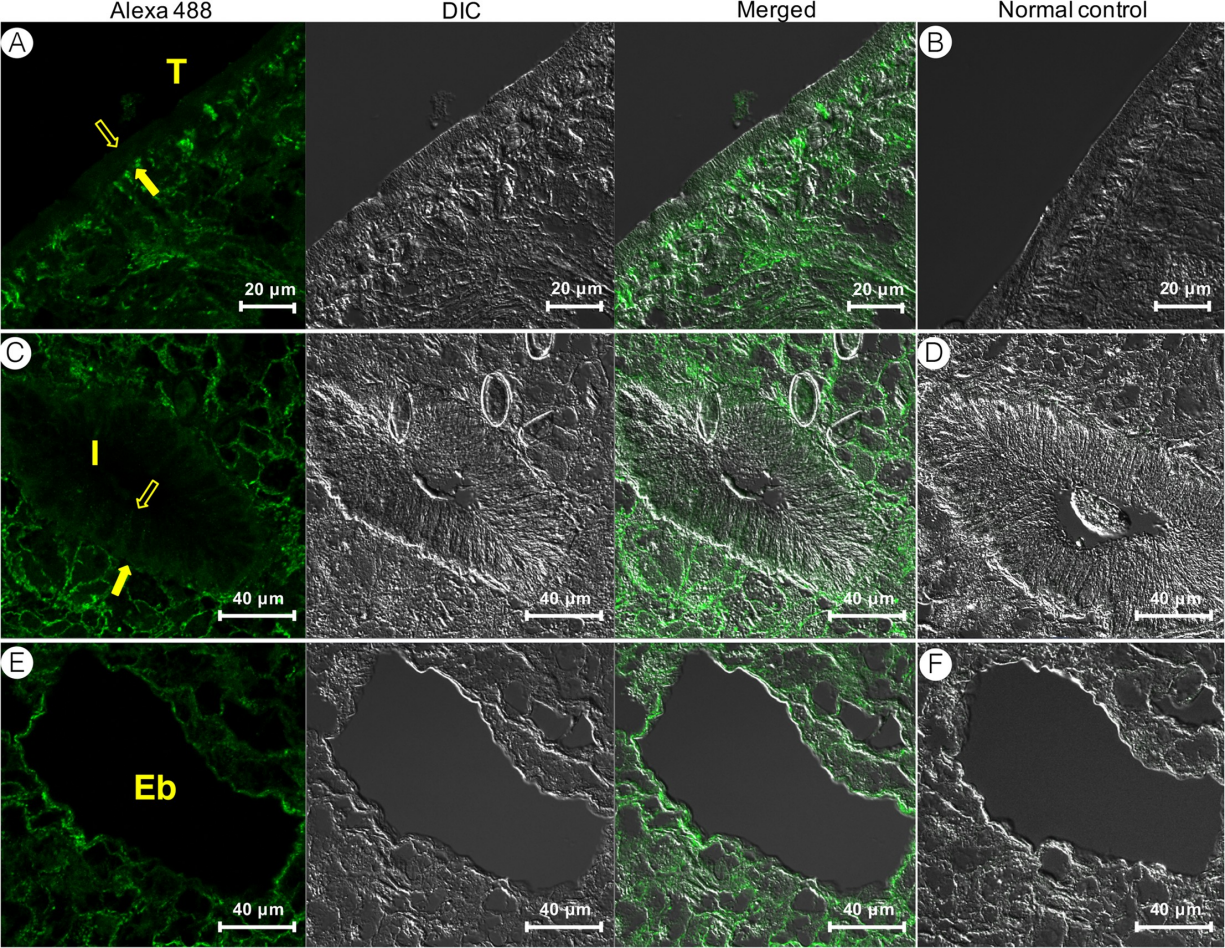
CsSBAT localization in *Clonorchis sinensis* adults using immunofluorescence staining. Left panels, mouse anti-CsSBAT immune serum; Right panels, normal mouse serum. A & B, tegument; C & D, intestine; E & F, excretory bladder. Open arrows indicate surface, and closed arrows indicate basement. Eb, excretory bladder; I, intestine; T, tegument.

In the *C*. *sinensis* adults, CsSBAT was abundant in the mesenchymal tissue throughout the whole body (Fig 5). In immunofluorescent staining, CsSBAT was localized in the mesenchymal tissue, basal layer of the tegument, intestinal ceca, and excretory bladder wall, but not in the surface membrane of the tegument and intestine (Fig 6).

### Interference with CsSBAT mRNA accelerated *C*. *sinensis* death in bile

**Fig 7:**
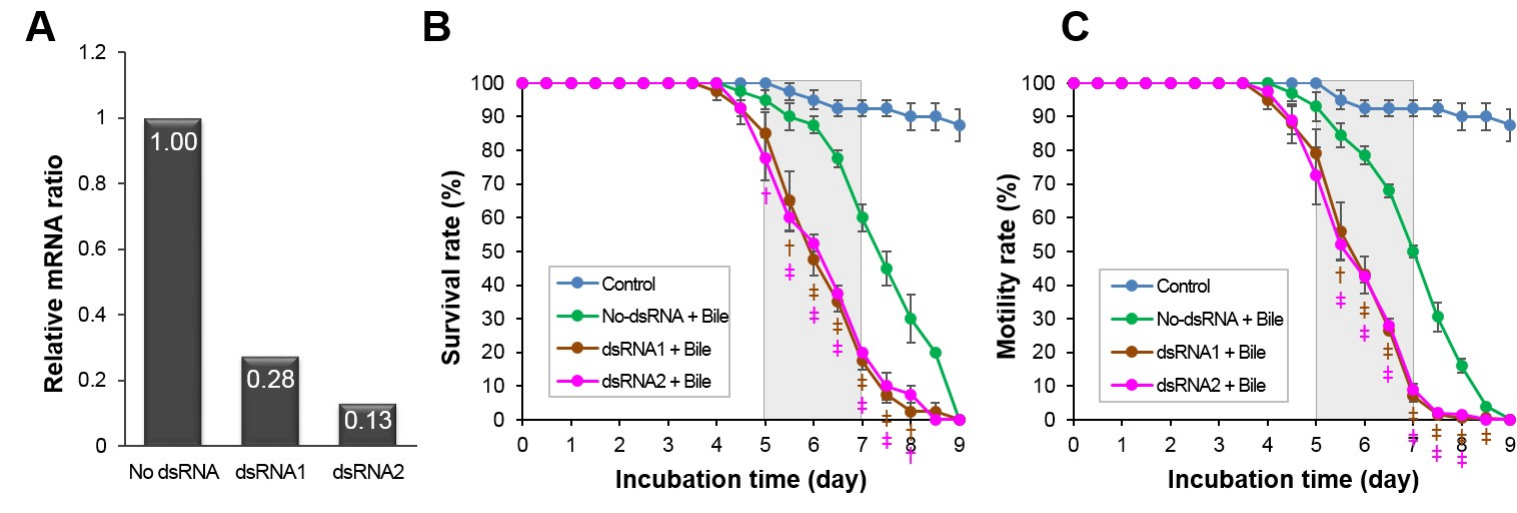
Effect of *CsSBAT* silencing using RNAi. (A) Downregulation of *CsSBAT* transcripts measured using RT-qPCR. No dsRNA, flukes incubated without dsRNA; dsRNA1 and dsRNA2, flukes incubated in CsSBAT-dsRNA1 or CsSBAT-dsRNA2. (B) Survival and (C) motility of *Clonorchis sinensis* adults incubated in CsSBAT-dsRNA1 or CsSBAT-dsRNA2 plus 0.075% bile solution. In control groups, *C*. *sinensis* were incubated in 1× Locke’s solution or plus 0.075% bile solution. Data represent mean ± standard error. ^†^*p* < 0.05, ^‡^*p* < 0.01, compared to flukes in the bile only.

Double-stranded RNA fragments of 259 bp (CsSBAT-dsRNA1) and 214 bp (CsSBAT-dsRNA2) were synthesized (S3 Fig). When soaked in the RNAi solutions for 6 h, the *CsSBAT* transcripts in the *C*. *sinensis* adults reduced significantly by 72% in dsRNA1 and by 87% in dsRNA2 solutions (Fig 7A).

Survival and motility of *C*. *sinensis* adults were assayed in 0.075% bile since they survived the longest in 0.005% bile, for two weeks in 0.05% bile, and for five days in 0.1% bile [54]. The robust silencing of *CsSBAT* transcripts greatly affected motility of the *C*. *sinensis* adults and deteriorated their survival compared to the CsSBAT-competent adults (Fig 7B and 7C). The adult flukes started to die from day 3.5 onward in the dsRNA1 group and from day 4 onward in the dsRNA2 group. Furthermore, there was a significant increase in fluke death from day 5 onward, and all flukes died by day 9 in both the dsRNA solutions.

The activity of flukes soaked in both the dsRNA1 and dsRNA2 solutions decreased rapidly from day 4 onward. More than 80% of flukes died on day 7, and the living ones showed little motility, while the majority of flukes without dsRNA treatment still showed motility in the 0.075% bile solution (Fig 7C).

### CsSBAT inhibition accelerates *C*. *sinensis* adult death

**Fig 8:**
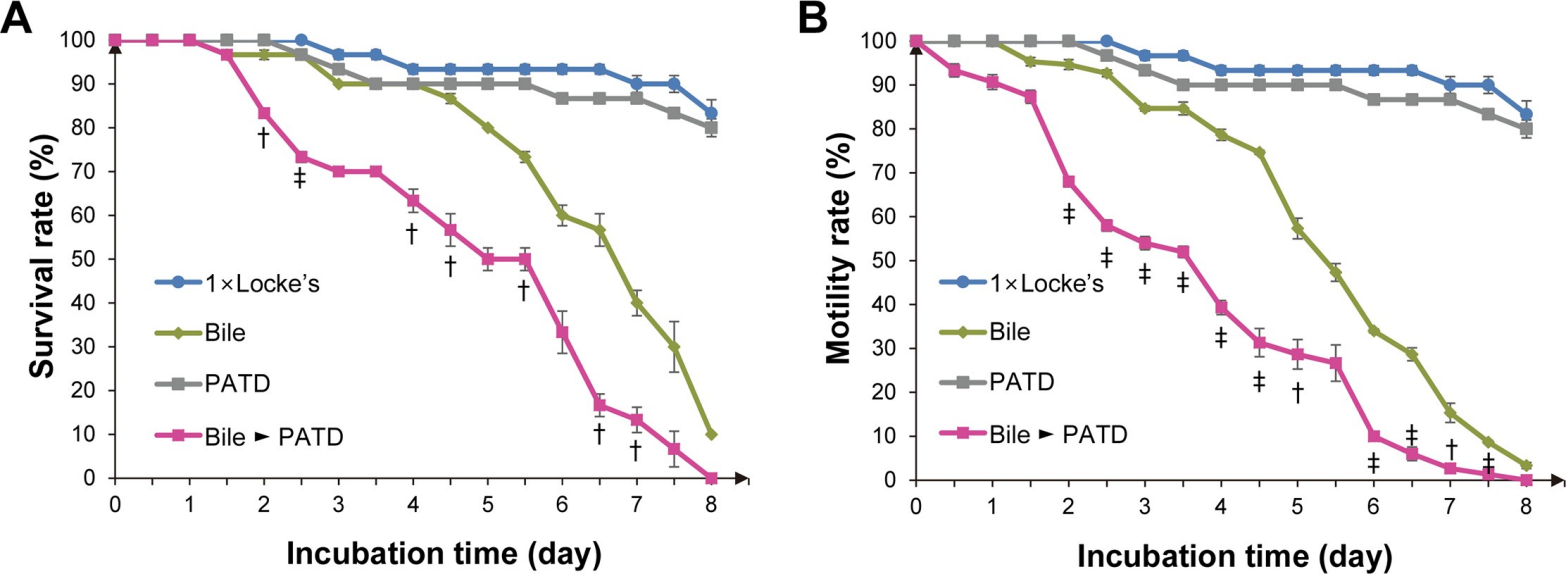
Survival and motility of *C*. *sinensis* adults in the bile with polyacrylic acid–tetradeoxycholic acid conjugates (PATD). (A) Survival curve. (B) Motility curve. Bile concentration, 0.075%; PATD concentration, 100 μg/mL. Data represent mean ± standard error. ^†^*p* < 0.05 and ^‡^*p* < 0.01 compared to the bile group.

PATD was used to inhibit CsSBAT [55]. PATD at 100 μg/mL had negligible influence on *C*. *sinensis* adults in 1× Locke’s solution without bile. During the course of the assay, the *C*. *sinensis* adults in the control and PATD groups survived for a long time and maintained active motility. The flukes in the bile (0.075%) group gradually died from day 3 onward after incubation. In contrast, the flukes in the experimental group (bile + PATD) died more quickly from day 2 onward (Fig 8A). The activity of living flukes in the experimental group also significantly decreased (Fig 8B).

When CsSBAT was inhibited using PATD, the survival rate of flukes started to decline 2 days earlier than that of flukes *CsSBAT*-silenced using RNAi (Figs 7B and 8A). This can be because PATD immediately clogged and inhibited CsSBAT, while *CsSBAT* RNAi suppressed SBAT synthesis and the cell membrane supply, resulting in delayed decrease of the overall function along with a decrease in density. Nevertheless, these results clearly showed that CsSBAT is crucial for *C*. *sinensis* adults to survive in the bile.

### CsSBAT inhibition led to accumulation of bile acids in *C*. *sinensis*

To understand why CsSBAT inhibition triggered rapid death of the *C*. *sinensis* adults compared to normal flukes, movement of fluorescence-labeled bile acids in the *C*. *sinensis* adults was observed by confocal microscopy. Lipofuscin, a heterogeneous fluorescent pigment [67], is abundant in *C*. *sinensis* adults. The older the flukes, the more lipofuscin they accumulate [68]. To minimize the autofluorescence, we used 15-day-old *C*. *sinensis* flukes [69] for confocal microscopy, which did not exhibit a visible background fluorescence.

A fluorescent bile acid derivative, tauro-nor-THCA-24-DBD, possesses transport features like taurocholic acid. This was employed in our study to observe bile acid distribution in the *C*. *sinensis*. When exposed to 1–500 μM of tauro-nor-THCA-24-DBD for 10 min, fluorescence intensity (FI) in the *C*. *sinensis* adults increased in a concentration-dependent manner up to 125 μM (S13A and S13B Fig). FI increased and saturated in 5 min post-incubation and remained at a plateau in 5 and 25 μM of tauro-nor-THCA-24-DBD. The FI difference between the two concentrations was not significant (S13C Fig). Accordingly, the CsSBAT inhibition effect was measured using 25 μM tauro-nor-THCA-24-DBD solution for 5 min.

**Fig 9:**
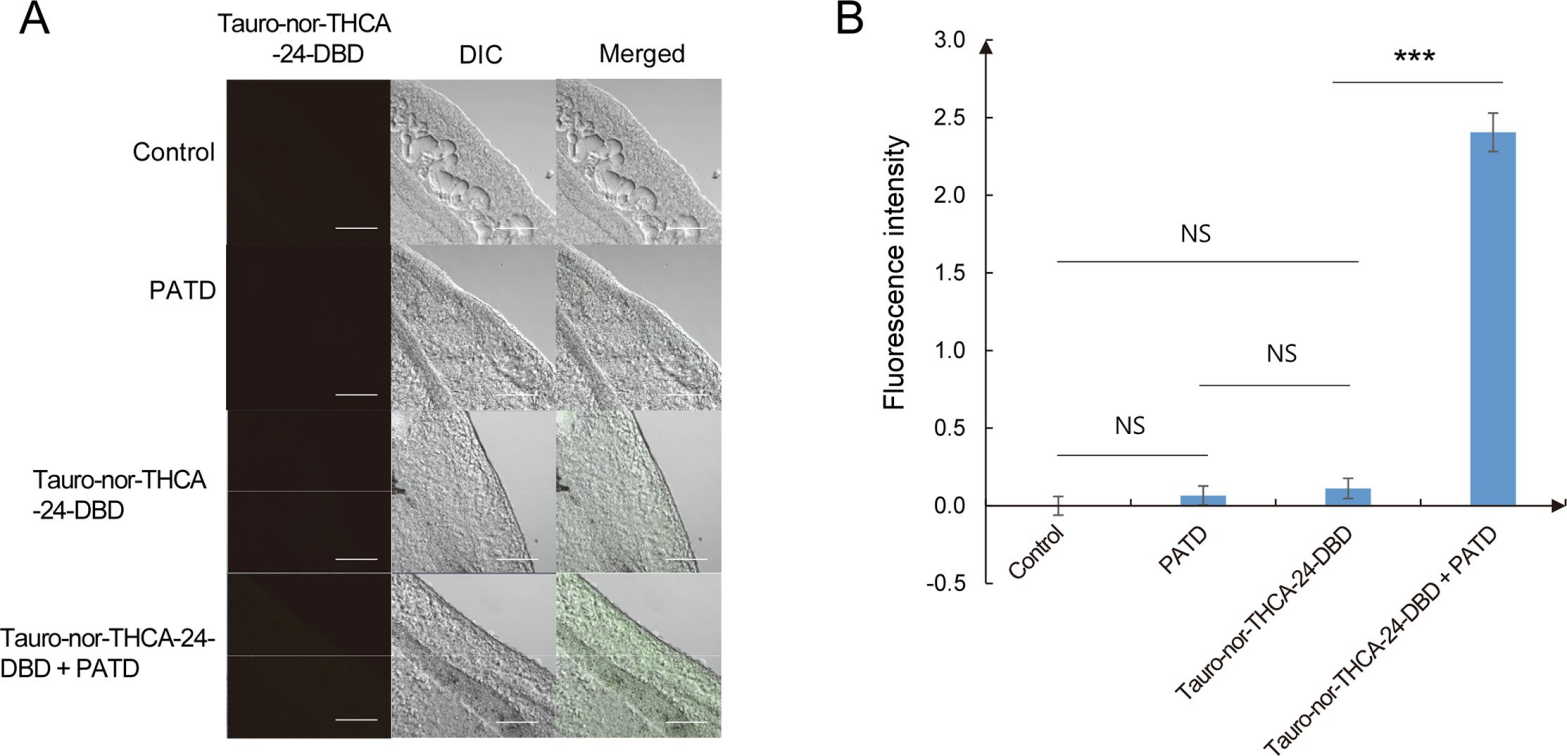
CsSBAT inhibition with polyacrylic acid–tetradeoxycholic acid conjugate (PATD) and bile acid accumulation. (A) Confocal micrographs on the lateral region of intestine bifurcation in the *Clonorchis sinensis* adults. Scale bar = 80 μm. (B) Inhibitory effect of PATD on CsSBAT presented as fluorescence intensity of tauro-nor-THCA-24-DBD accumulated in the mesenchymal tissue. Tauro-nor-THCA-24-DBD concentration, 25 μM; PATD concentration, 400 μg/mL. Bar represents mean ± standard error. ***p < 0.001; NS, not significant.

The *C*. *sinensis* adults in the tauro-nor-THCA-24-DBD group revealed weak FI that did not significantly differ from that of the controls. In contrast, the FI of the tauro-nor-THCA-24-DBD plus PATD group was significantly stronger than that of the other groups (*p* < 0.001) (Fig 9A and 9B). These results suggest that the *C*. *sinensis* adult maintains bile acids at a low concentration under its normal physiological condition. The bile acid transportation was impeded when the CsSBAT function was suppressed, resulting in bile acid accumulation in the mesenchymal tissue. The accumulated bile acids could disturb physiologic homeostasis and reduce survival of the *C*. *sinensis* adults.

## Discussion

Sodium-bile acid co-transporter (SBAT) is the second member of the SLC10 family (SLC10A2). In higher animals, it is mainly localized on the apex of enterocytes in the distal ileum. Hence, it is also referred to as ileal bile salt transporter or ASBT [70,71]. However, there are few studies on its function in platyhelminths. The *SLC10* gene was identified from *Taenia solium* [72] and BSEP from *Fasciola gigantica* [73] without functional studies. Here, we identified the first SBAT protein, CsSBAT, in trematodes. The abbreviation CsSBAT was given based on three facts. First, CsSBAT had all conserved and functional residues of HsASBT and HsNTCP, implying structural and functional conservation. Second, CsSBAT shared higher sequence homology with HsASBT than HsNTCP. Finally, its tissue distribution was basal/inward rather than apical/outward. In addition, CsSBAT had N- and C-terminal disordered regions, which were approximately 237 amino acids longer than mammalian SBATs. These two regions were specific to the trematodes and might attribute to the different CsSBAT tissue distributions in the *C*. *sinensis* adults.

Discontinuous TM helices are observed in several secondary active transporters such as Na^+^/H^+^ antiporter and leucine transporter [74]. ASBTs showed a topology like the Na^+^/H^+^ antiporter structure, but they had low sequence homology. The TM5 was highly conserved across the eukaryotic ASBT homologs. Its residues interacted spatially with residues in the proximity of neighboring TMs, especially with TM6, conveying helical flexibility [21].

Bile components are prerequisite as chemotropic attractants to trigger the migration of juvenile *C*. *sinensis* toward the bile duct [75]. Bile components trigger metabolic pathways and enhance endoparasite motility [76]. The juvenile *C*. *sinensis* is expected to utilize bile acids for their physico-metabolism and residence in mammalian host bile ducts. CsSBAT mRNA was more abundant in the metacercariae than in the adults. In the mammalian hosts, the *C*. *sinensis* metacercariae excyst in the duodenum upon meeting the bile and the newly excysted juveniles migrate into the bile duct [6]. To adapt immediately to the overwhelming bile shock, the metacercariae have to be prepared and contain high levels of all the molecular instruments before encountering the bile. Therefore, the *C*. *sinensis* metacercariae have more bile transporters to manage the bile than the adults that have adapted to the bile environment and maintained bile homeostasis.

However, when bile components are excessive in the body, they can impose toxic effects and damage the cells and tissues both of the *C*. *sinensis* metacercariae and adults. Bile acids decrease locomotive cycles of the juvenile *F*. *hepatica* and cause death [77]. High bile concentrations accelerated the death of both juvenile and adult *C*. *sinensis* [54,75]. As a bile transporter, CsSBAT was expected to be crucial for bile transportation and detoxification in *C*. *sinensis*.

CsSBAT was abundantly distributed in the mesenchymal tissues similar to other bile transporters in trematodes. In *F*. *hepatica*, two bile salt transporters, BSEP and MRP1, were distributed in the mesenchymal tissue and tegument basal layer, suggesting that they might regulate chemical diffusion in this fluke [73]. In *C*. *sinensis*, CsMRP4 and CsOST were distributed in the mesenchymal tissue and proposed to pump bile salts out of the cells [27,28]. The CsSBAT was colocalized with CsOST and CsMRP4 in the mesenchymal tissue of *C*. *sinensis* adults, similar to HsNTCP in the hepatocytes [11,12]. If CsSBAT is localized on the surface of the tegument and intestine of adult *C*. *sinensis* similar to HsASBT at the enterocyte apex of the human ileal epithelium [11,12], it inevitably encounters high concentration gradients of bile acids and sodium ions, because the fluke lives immerged in bile and engulfs it in the intestine [6]. Under this situation, bile acids can flood through CsSBAT and overwhelm the physiologic homeostasis, which deteriorates *C*. *sinensis*. However, *C*. *sinensis* evaded detriment because CsSBAT is localized under the tegument basal layer and not on the superficial membrane as evidenced by our immunohistochemical and immunofluorescence confocal findings. As for bile acid permeation, bile acid (measured as tauro-nor-THCA-24-DBD) increased for the first 5 min before reaching a saturation level as indicated by the low fluorescence intensity. It is assumed that CsSBAT imports bile acids from the mesenchymal space into the cells, while CsMRP4 and CsOST pump out bile acids from the cells.

In the bile ducts, *C*. *sinensis* regulates bile acids at normal physiological levels favorable for metabolism. However, when CsSBAT was inhibited by PATD, bile acids significantly accumulated in the *C*. *sinensis* adults incubated in bile, accelerating their deaths. This also occurred when *CsSBAT* expression was efficiently silenced with dsRNA1 and dsRNA2. CsSBAT is unusually longer than other SBATs, with unique extensions of approximately 200 amino acids at both the N- and C-termini. Our RNAi targeting the unique region in the CsSBAT mRNA was effective and knocked down gene expression.

The *C*. *sinensis* mesenchymal cells are equipped with three bile acid transporters: CsSBAT importer and CsOST and CsMRP4 exporters [27,28]. In the *C*. *sinensis* adults, bile acids were likely to be transported in and out of the cells via these transporters across the mesenchymal tissues and presumably into the excretory bladder. Meanwhile, CsSBAT repression using RNAi or PATD could cause bile acid stagnation/accumulation in the mesenchymal tissues, which deteriorated *C*. *sinensis* adult survival and led to death in the bile. It is not clear whether the mesenchymal cells have a polarity oriented posteriorly to aid the bile acid transportation in the *C*. *sinensis*. In the liver, the hepatocytes maintain a polarity to allow bile salt transportation, *i*.*e*., they import bile acids through HsNTCP and export them into the bile canaliculi via the exporters [12,13]. As for *C*. *sinensis*, the mechanism of the bile salt transportation through the mesenchymal cells deserves further studies. The tight inhibitors to the CsSBAT could be exploited to explore and develop anthelminthic drugs against liver flukes inhabiting the bile.

Taken together, we identified an SBAT (CsSBAT) in the *C*. *sinensis* adults and characterized its molecular biological features, 3D structure, and distribution in the tissues of the body. CsSBAT repression using inhibitor and RNAi was detrimental to the *C*. *sinensis* adults, suggesting that CsSBAT is a bile acid transporter crucial for *C*. *sinensis* survival in the bile duct.

## Supporting information

S1 Fig Construction of antigenic chimera of CsSBAT. Putative B-cell epitopes and hydrophilic regions were predicted on CsSBAT (pink box). The epitope regions were each PCR-amplified and subcloned into an expression plasmid vector. A spacer peptide, GPGPG, was inserted between the two epitopes. For details, refer to Materials and methods.
(TIF)

S2 Fig cDNA and deduced amino acid of CsSBAT-chimeric polypeptide sequences. A spacer peptide GPGPG is highlighted with yellow background. Blue backgrounds indicate restriction enzyme sites (*Bam*HI, *Eco*RI, and *Hin*dIII).
(TIF)

S3 Fig CsSBAT*-*dsRNA1 and CsSBAT-dsRNA2. (A) Selection of dsRNA-targeted regions in CsSBAT. Blue bar indicates the highly conserved region of CsSBAT in other species. Green bars show the extra sequences that do not exist in other SBATs. The green striped bars indicate finally selected regions for dsRNA synthesis specific to CsSBAT. (B) Purified T7 promoter-tagged DNA templates for CsSBAT-dsRNA1 and dsRNA2 synthesis.
(TIF)

S4 Fig cDNA and putative polypeptide sequences of CsSBAT. (TIF)

S5 Fig Phylogenetic tree of CsSBAT with homologs and SLC10A family members. Homologous proteins and members of SLC10A family were retrieved from UniProtKB/Swiss-Prot database [34]. The evolutionary relationship was inferred using MEGA7. Node values were calculated using a bootstrap test (1,000 replicates) with the maximum-likelihood method. Cs, *Clonorchis sinensis*; Eg, *Echinococcus granulosus*; Em, *Echinococcus multilocularis*; Hs, *Homo sapiens*; Mm, *Mus musculus*; Nm, *Neisseria meningitidis*; Ov, *Opisthorchis viverrini*; Pw, *Paragonimus westermani*; Sm, *Schistosoma mansoni*; Yf, *Yersinia frederiksenii*.
(TIF)

S6 Fig CsSBAT disorder profile. Disordered regions were predicted on residues 1–184 and 494–546.
(TIF)

S7 Fig Validation of the final 3D model of inward facing-CsSBAT. (A) Ramachandran plot showing that the proportion of amino residues in the most favored regions is 92.5% and is 6.3% in the additionally allowed regions. The proportion of amino residues in the generously allowed regions is 0% and that in the disallowed regions is 1.2%. (B) ProSA energy profile showing Z-score of −3.73. (C) ERRAT plot indicating overall quality factor is 97.7%.
(TIF)

S8 Fig Superimposition of the structural repeats. (A) The first repeat consisted of TMs 1–5. (B) The second repeat consisted of TMs 6–10. (C) The first repeat was superimposed on the second repeat using iPBA (improved Protein Block Alignment) which shows a RMSD of 2.79 Å for 105 out of 203 pairs of Cα positions. (D) TMs 1–2 and TMs 6–7, forming panel domain, are aligned with each other. The superimposition shows a RMSD of 2.68 Å for 27 out of 94 pairs of Cα positions. (E) TMs 3–5 and TMs 8–10, forming core domain, are aligned with each other. The superimposition shows a RMSD of 2.58 Å for 69 out of 118 pairs of Cα positions.
(TIF)

S9 Fig Evolutionary conservation of CsSBAT. Ribbon model of CsSBAT is visualized in the membrane plane with the extracellular side up. Amino acids are colored by their conservation grades using the color-coding bar. Overall, TMs 4, 5, and 9 (boxed with broken line) are highly conserved, with average conservation scores of 8–9.
(TIF)

S10 Fig Developmental *CsSBAT* expression. Relative expression level of *CsSBAT* gene in metacercaria and adult stage of *C*. *sinensis* was measured using RT-qPCR. For methods, refer to section Materials and methods in the main text.
(TIF)

S11 Fig cDNAs synthesized to produce a chimeric antigenic protein of CsSBAT. PCR-amplified cDNA fragments of CsSBAT-cp1 (A) and CsSBAT-cp2 (B). (C) A connected cDNA of CsSBAT-cp1 and -cp2 was popped out by restriction double-digestion from an expression plasmid pRSET A.
(TIF)

S12 Fig Induction and purification of recombinant CsSBAT-chimeric protein. *E*. *coli* BL21[DE3]pLysS were transformed with plasmid DNA containing CsSBAT-chimeric cDNA. Recombinant CsSBAT-chimeric protein was induced by adding IPTG (isopropyl β-d-1-thiogalactopyranoside) into culture medium. The chimeric protein was purified using Ni-NTA column under denaturing condition. Un, uninduced total lysate; In, induced total lysate; CL, urea-treated clear supernatant; PT, pellet; W, the last washing; Eluate 1–6, the first to sixth fractions eluted from Ni-NTA column.
(TIF)

S13 Fig Tauro-nor-THCA-24-DBD permeation in *Clonorchis sinensis*. (A) Bile acid uptake in *C*. *sinensis* incubated in different tauro-nor-THCA-24-DBD concentrations. Scale bar = 80 μm. (B) Quantified fluorescence intensity based on panel (A). Fluorescence intensity of the negative control was subtracted from all groups. (C) Time course of bile acid permeation. No significant difference was observed between the two groups.
(TIF)
