# Supplementary figures and images for: Sodium-bile acid co-transporter is crucial for survival of a carcinogenic liver fluke Clonorchis sinensis in the bile
Source: PLoS Negl Trop Dis. 2020 Dec 7;14(12):e0008952. doi: 10.1371/journal.pntd.0008952 (PMC7746286; doi:10.1371/journal.pntd.0008952)

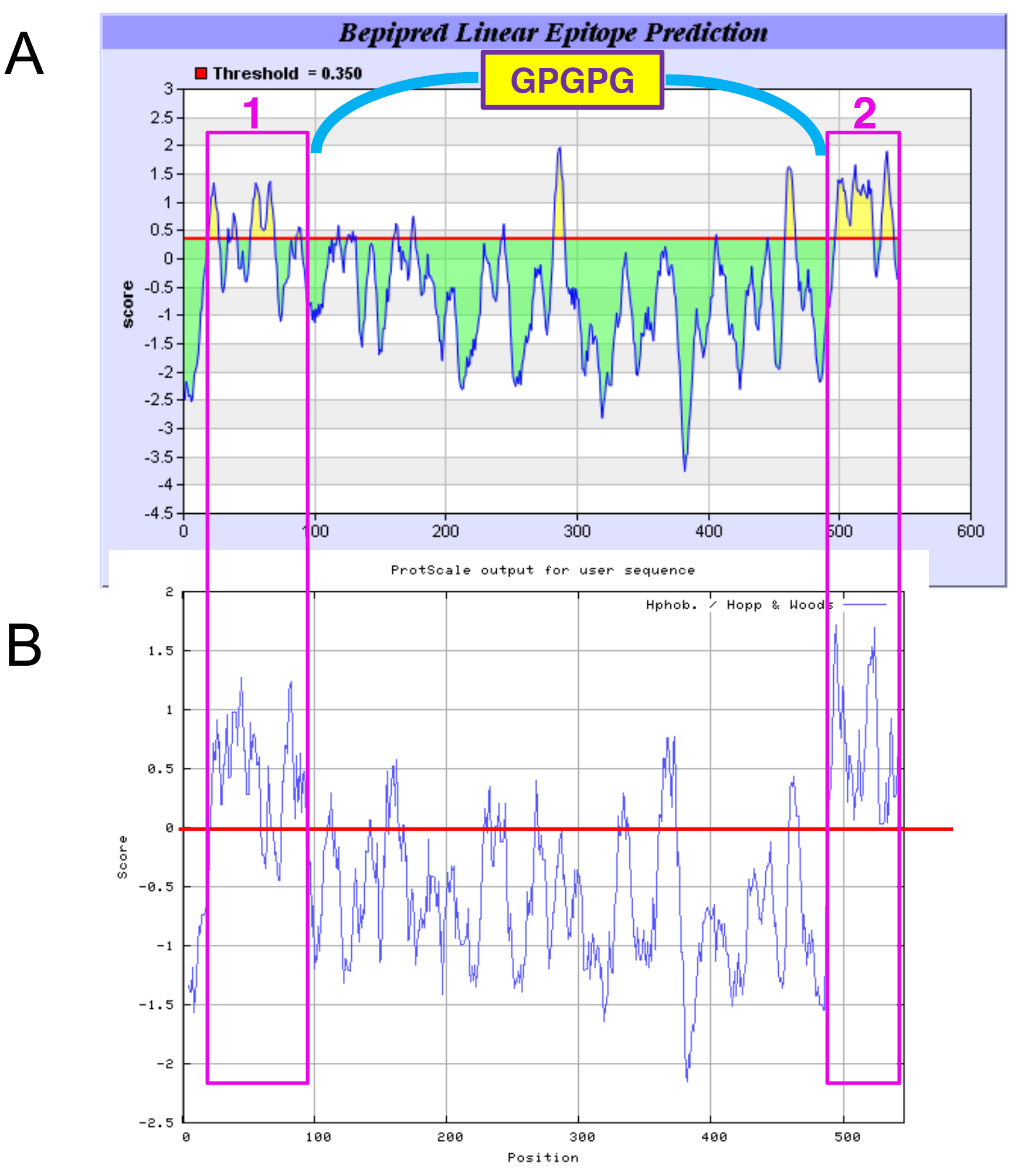

Supplement: S1 Fig — Putative B-cell epitopes and hydrophilic regions were predicted on CsSBAT (pink box). The epitope regions were each PCR-amplified and subcloned into an expression plasmid vector. A spacer peptide, GPGPG, was inserted between the two epitopes. For details, refer to Materials and methods. (TIF) [file pntd.0008952.s001.tif]

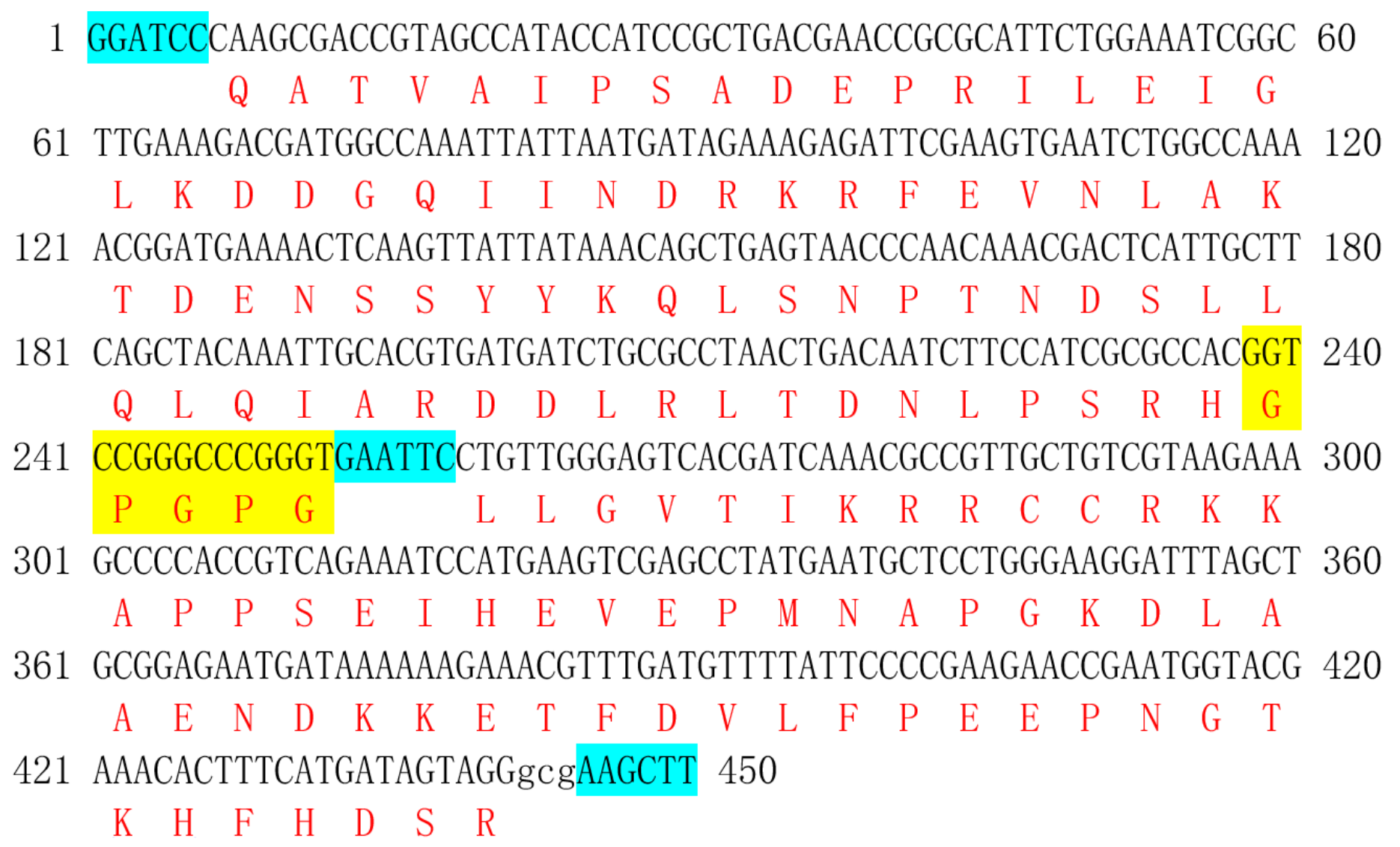

Supplement: S2 Fig — A spacer peptide GPGPG is highlighted with yellow background. Blue backgrounds indicate restriction enzyme sites (BamHI, EcoRI, and HindIII). (TIF) [file pntd.0008952.s002.tif]

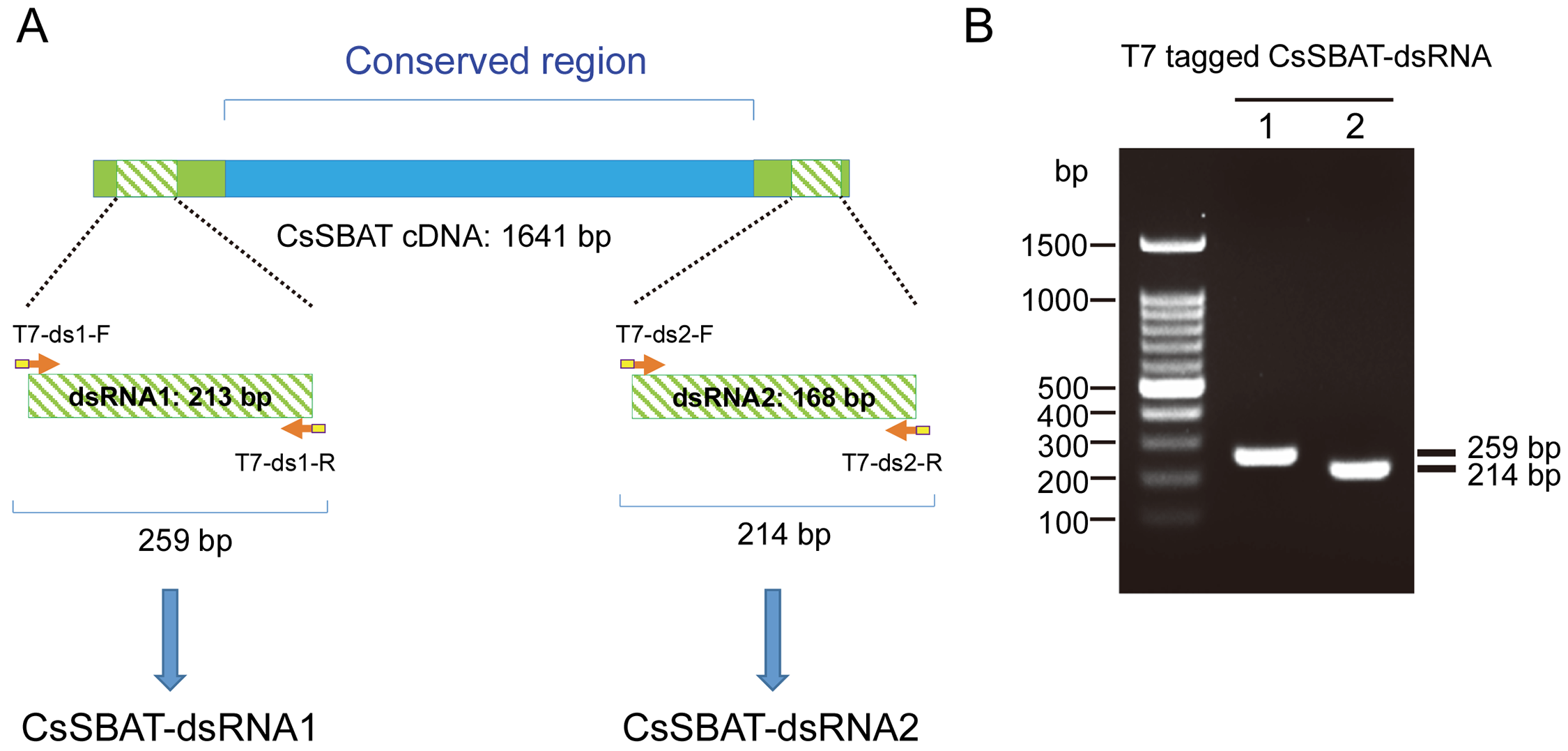

Supplement: S3 Fig — (A) Selection of dsRNA-targeted regions in CsSBAT. Blue bar indicates the highly conserved region of CsSBAT in other species. Green bars show the extra sequences that do not exist in other SBATs. The green striped bars indicate finally selected regions for dsRNA synthesis specific to CsSBAT. (B) Purified T7 promoter-tagged DNA templates for CsSBAT-dsRNA1 and dsRNA2 synthesis. (TIF) [file pntd.0008952.s003.tif]

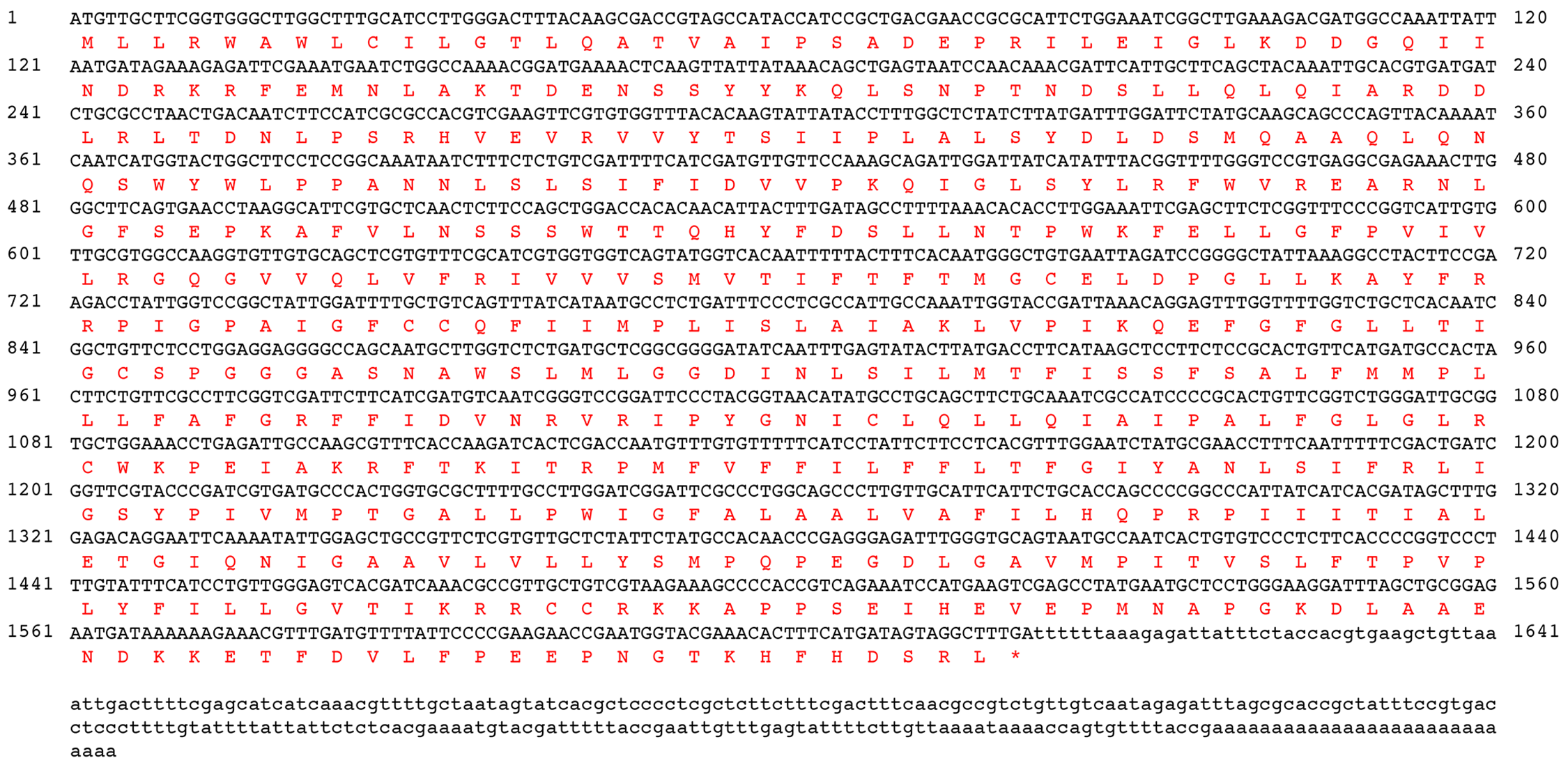

Supplement: S4 Fig — (TIF) [file pntd.0008952.s004.tif]

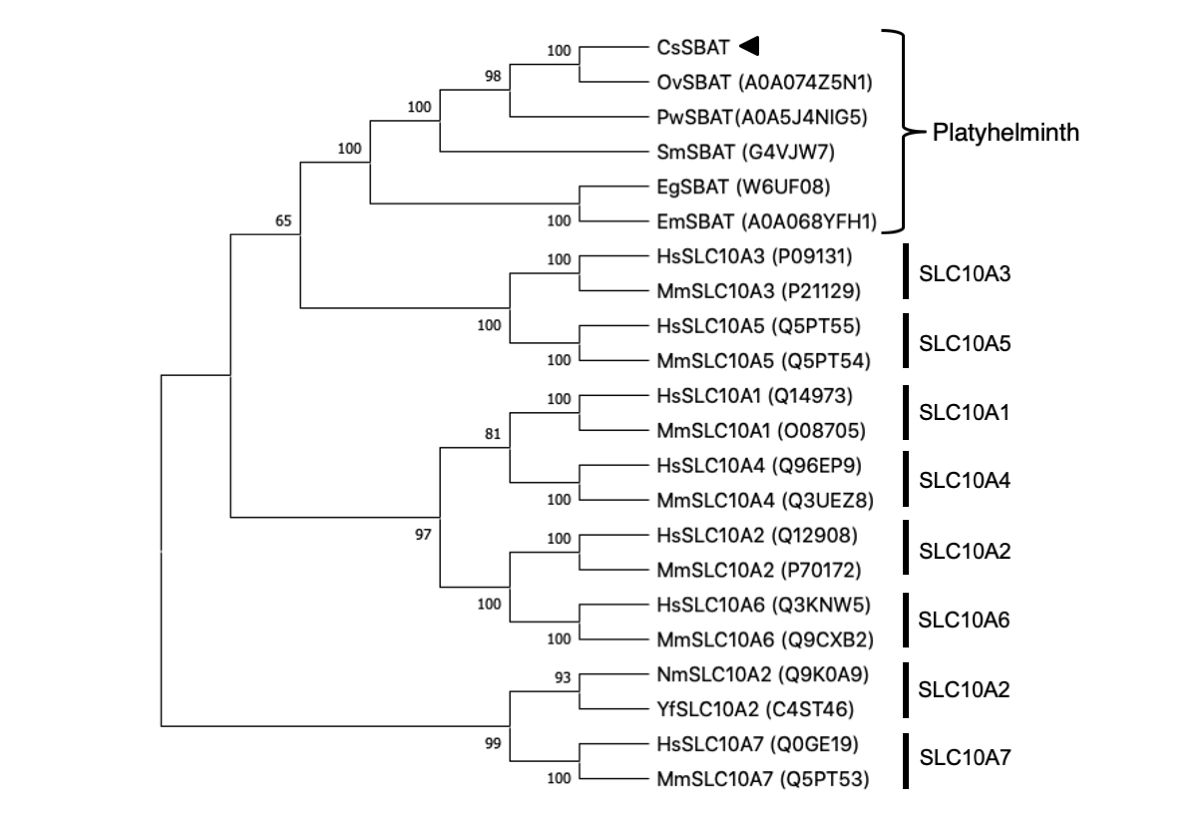

Supplement: S5 Fig — Homologous proteins and members of SLC10A family were retrieved from UniProtKB/Swiss-Prot database [34]. The evolutionary relationship was inferred using MEGA7. Node values were calculated using a bootstrap test (1,000 replicates) with the maximum-likelihood method. Cs, Clonorchis sinensis; Eg, Echinococcus granulosus; Em, Echinococcus multilocularis; Hs, Homo sapiens; Mm, Mus musculus; Nm, Neisseria meningitidis; Ov, Opisthorchis viverrini; Pw, Paragonimus westermani; Sm, Schistosoma mansoni; Yf, Yersinia frederiksenii. (TIF) [file pntd.0008952.s005.tif]

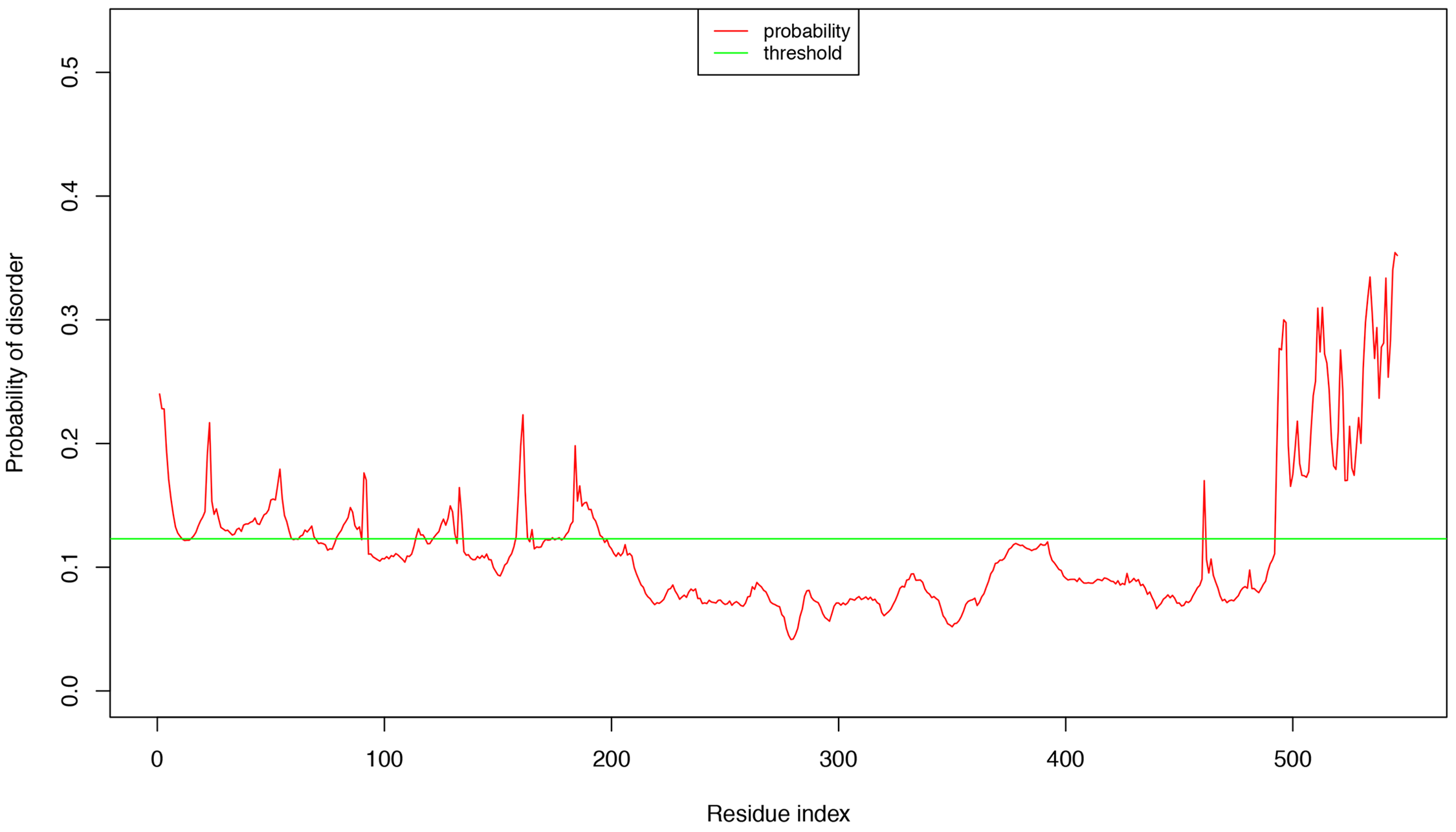

Supplement: S6 Fig — Disordered regions were predicted on residues 1–184 and 494–546. (TIF) [file pntd.0008952.s006.tif]

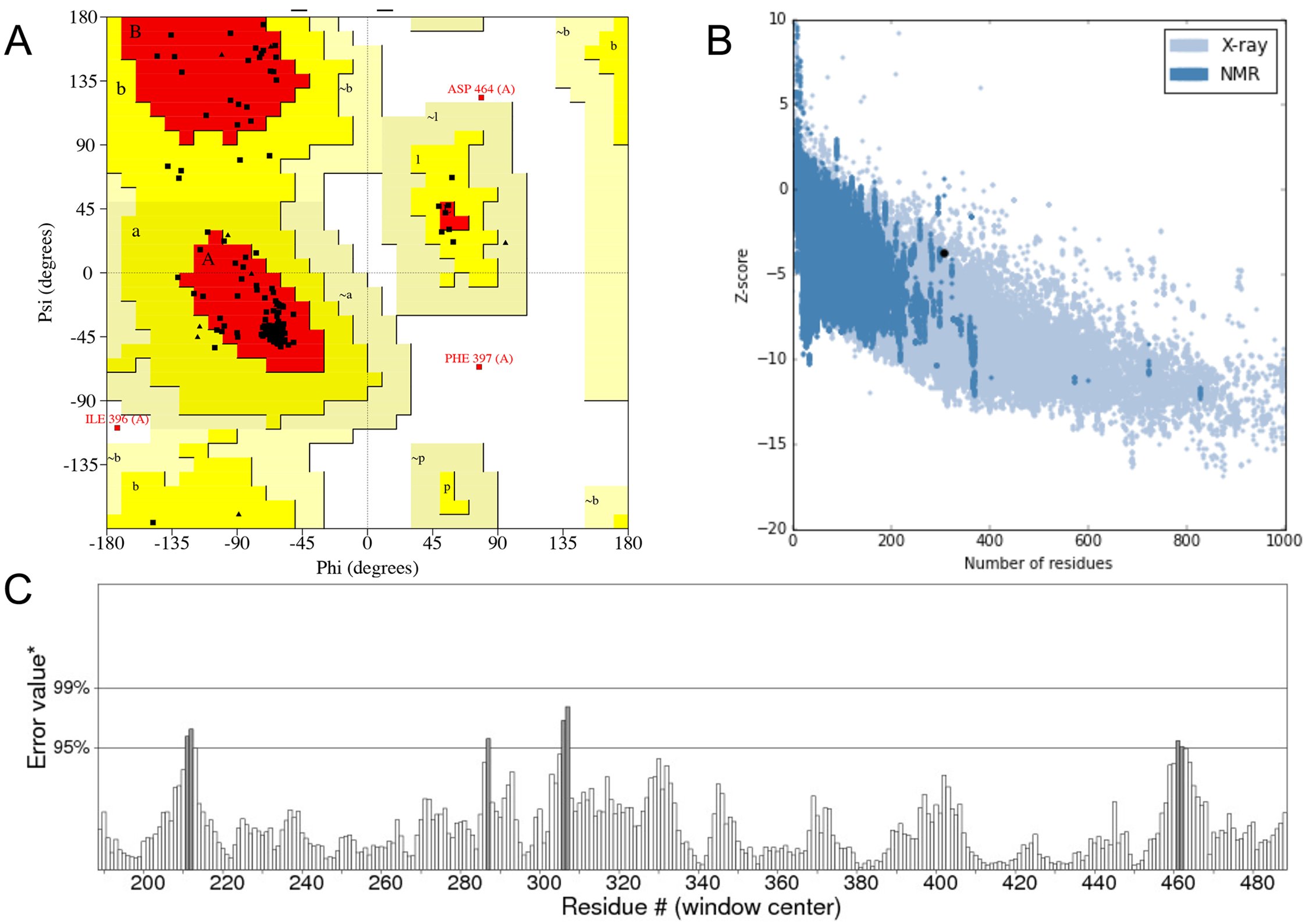

Supplement: S7 Fig — (A) Ramachandran plot showing that the proportion of amino residues in the most favored regions is 92.5% and is 6.3% in the additionally allowed regions. The proportion of amino residues in the generously allowed regions is 0% and that in the disallowed regions is 1.2%. (B) ProSA energy profile showing Z-score of −3.73. (C) ERRAT plot indicating overall quality factor is 97.7%. (TIF) [file pntd.0008952.s007.tif]

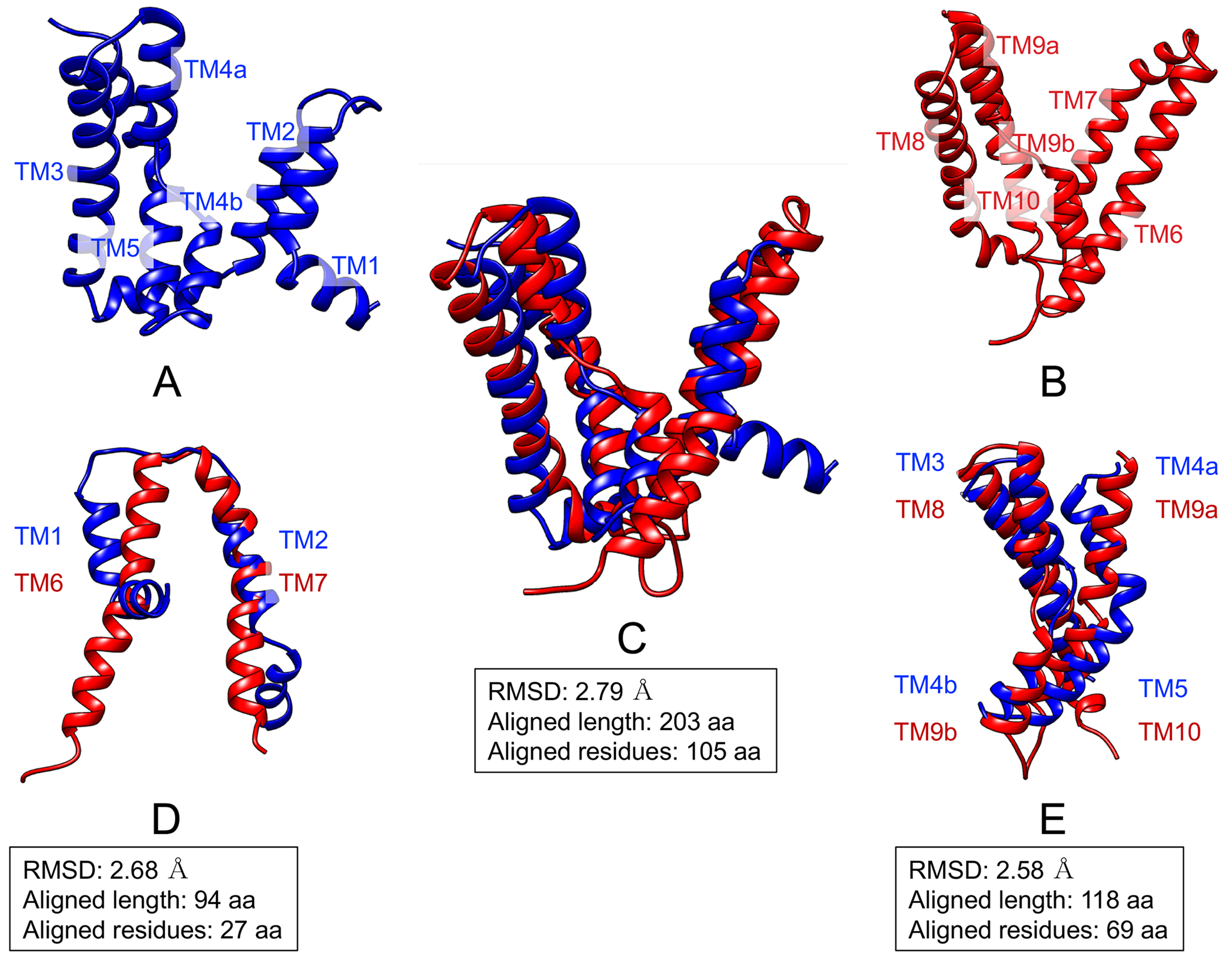

Supplement: S8 Fig — (A) The first repeat consisted of TMs 1–5. (B) The second repeat consisted of TMs 6–10. (C) The first repeat was superimposed on the second repeat using iPBA (improved Protein Block Alignment) which shows a RMSD of 2.79 Å for 105 out of 203 pairs of Cα positions. (D) TMs 1–2 and TMs 6–7, forming panel domain, are aligned with each other. The superimposition shows a RMSD of 2.68 Å for 27 out of 94 pairs of Cα positions. (E) TMs 3–5 and TMs 8–10, forming core domain, are aligned with each other. The superimposition shows a RMSD of 2.58 Å for 69 out of 118 pairs of Cα positions. (TIF) [file pntd.0008952.s008.tif]

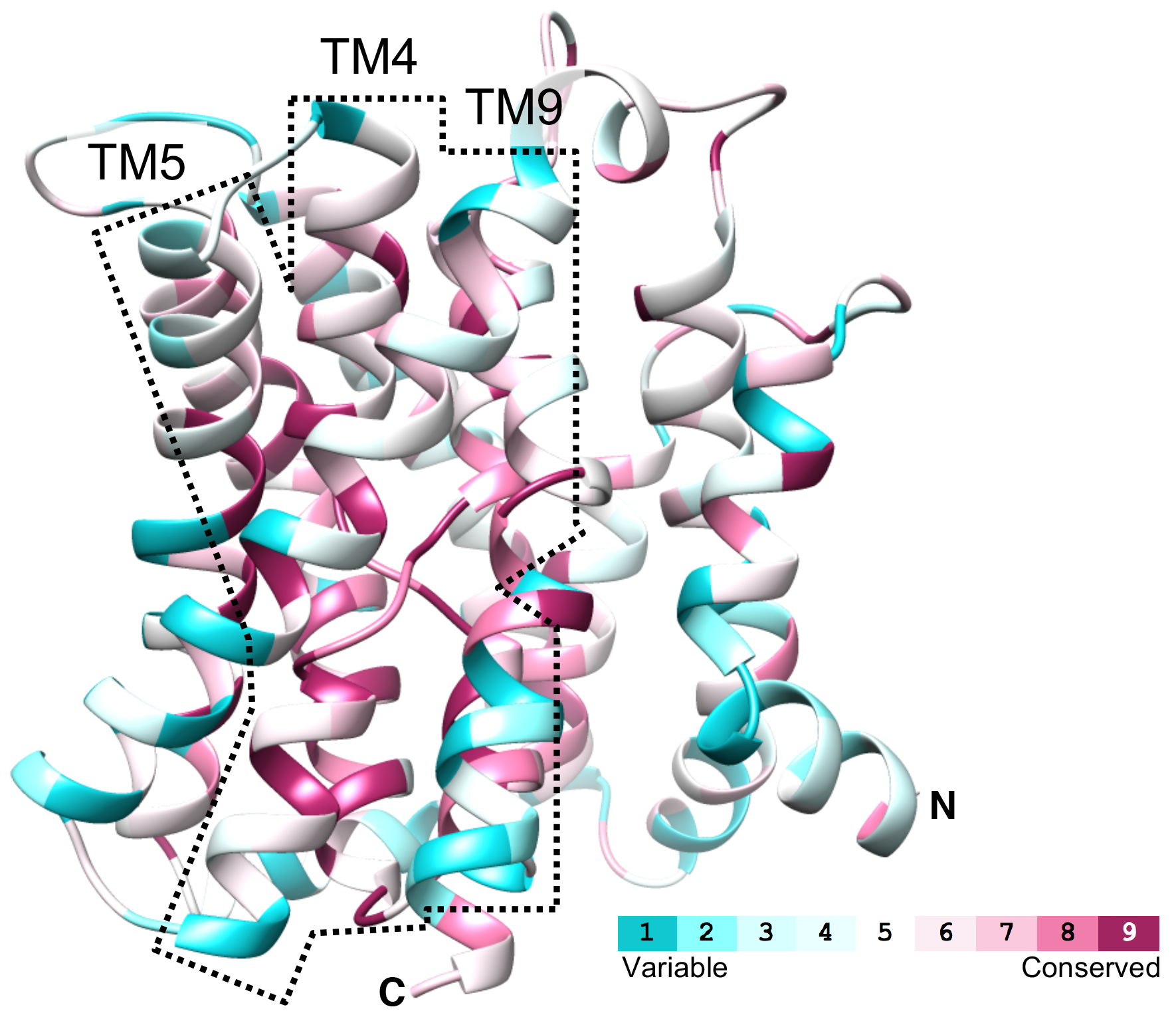

Supplement: S9 Fig — Ribbon model of CsSBAT is visualized in the membrane plane with the extracellular side up. Amino acids are colored by their conservation grades using the color-coding bar. Overall, TMs 4, 5, and 9 (boxed with broken line) are highly conserved, with average conservation scores of 8–9. (TIF) [file pntd.0008952.s009.tif]

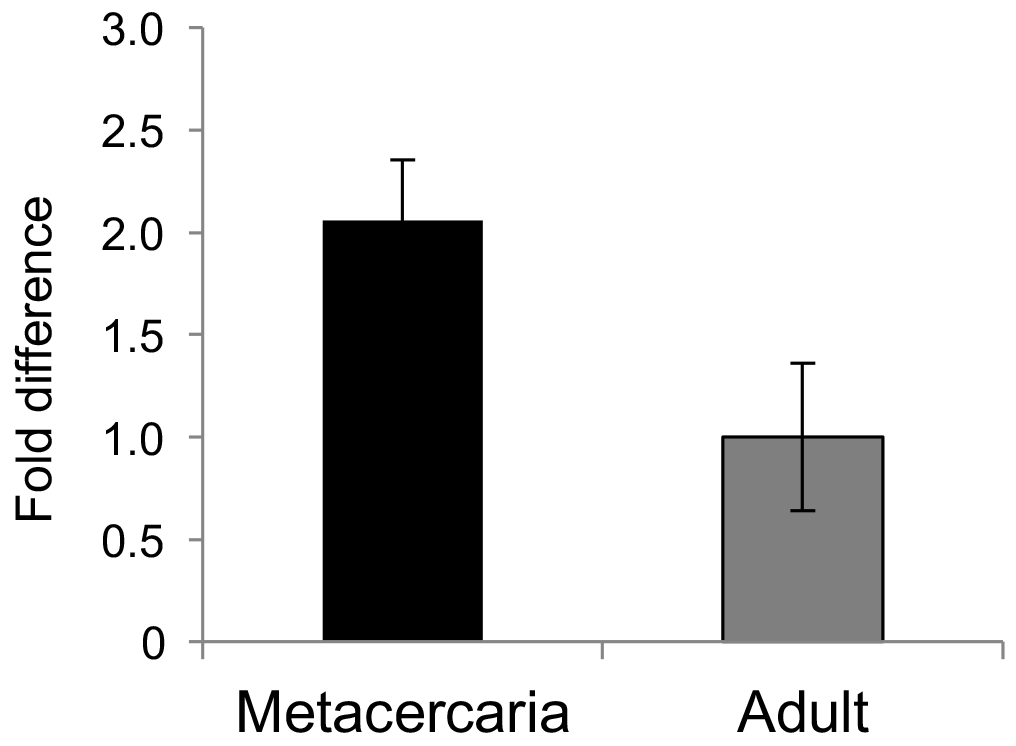

Supplement: S10 Fig — Relative expression level of CsSBAT gene in metacercaria and adult stage of C. sinensis was measured using RT-qPCR. For methods, refer to section Materials and methods in the main text. (TIF) [file pntd.0008952.s010.tif]

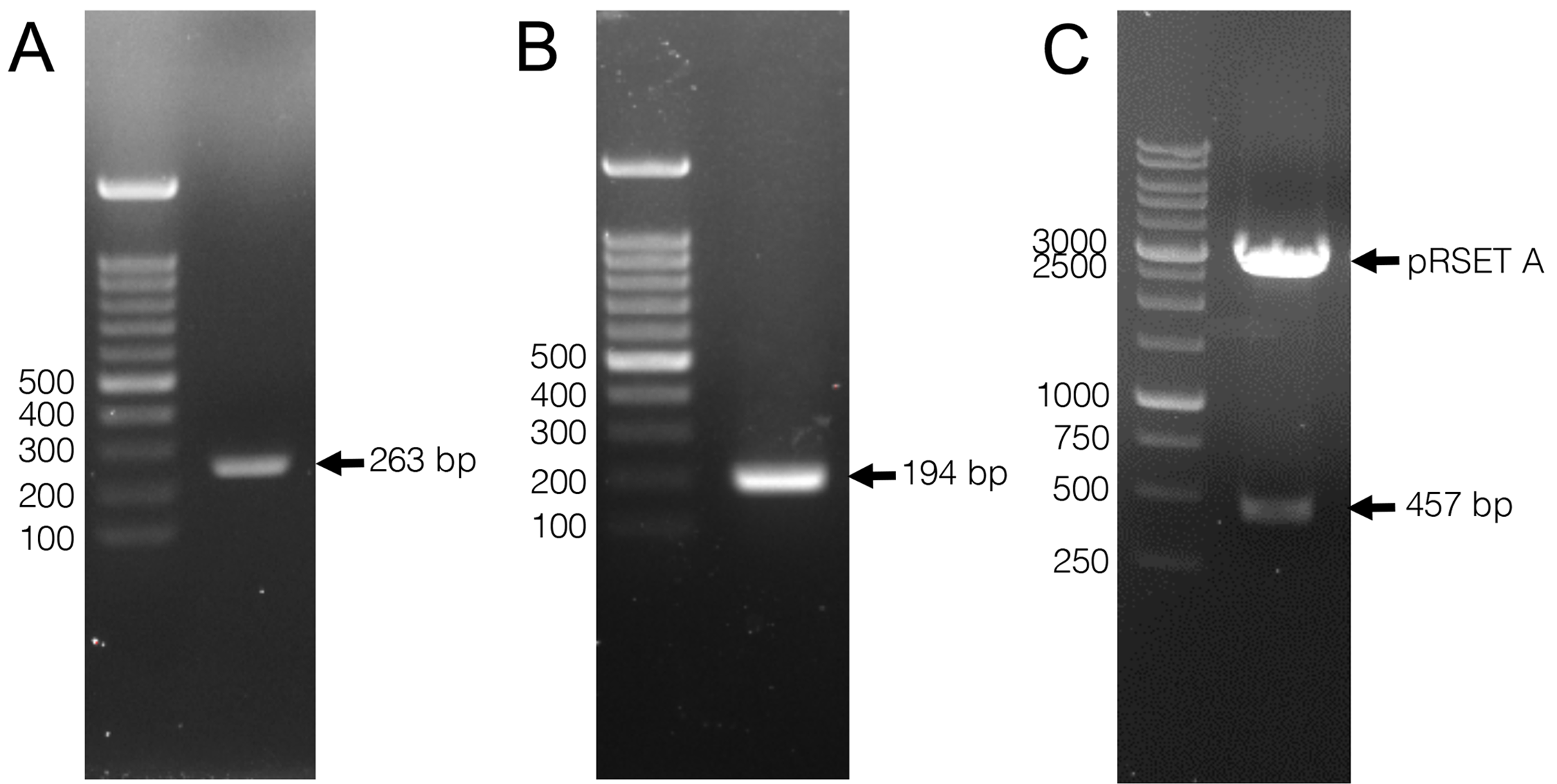

Supplement: S11 Fig — PCR-amplified cDNA fragments of CsSBAT-cp1 (A) and CsSBAT-cp2 (B). (C) A connected cDNA of CsSBAT-cp1 and -cp2 was popped out by restriction double-digestion from an expression plasmid pRSET A. (TIF) [file pntd.0008952.s011.tif]

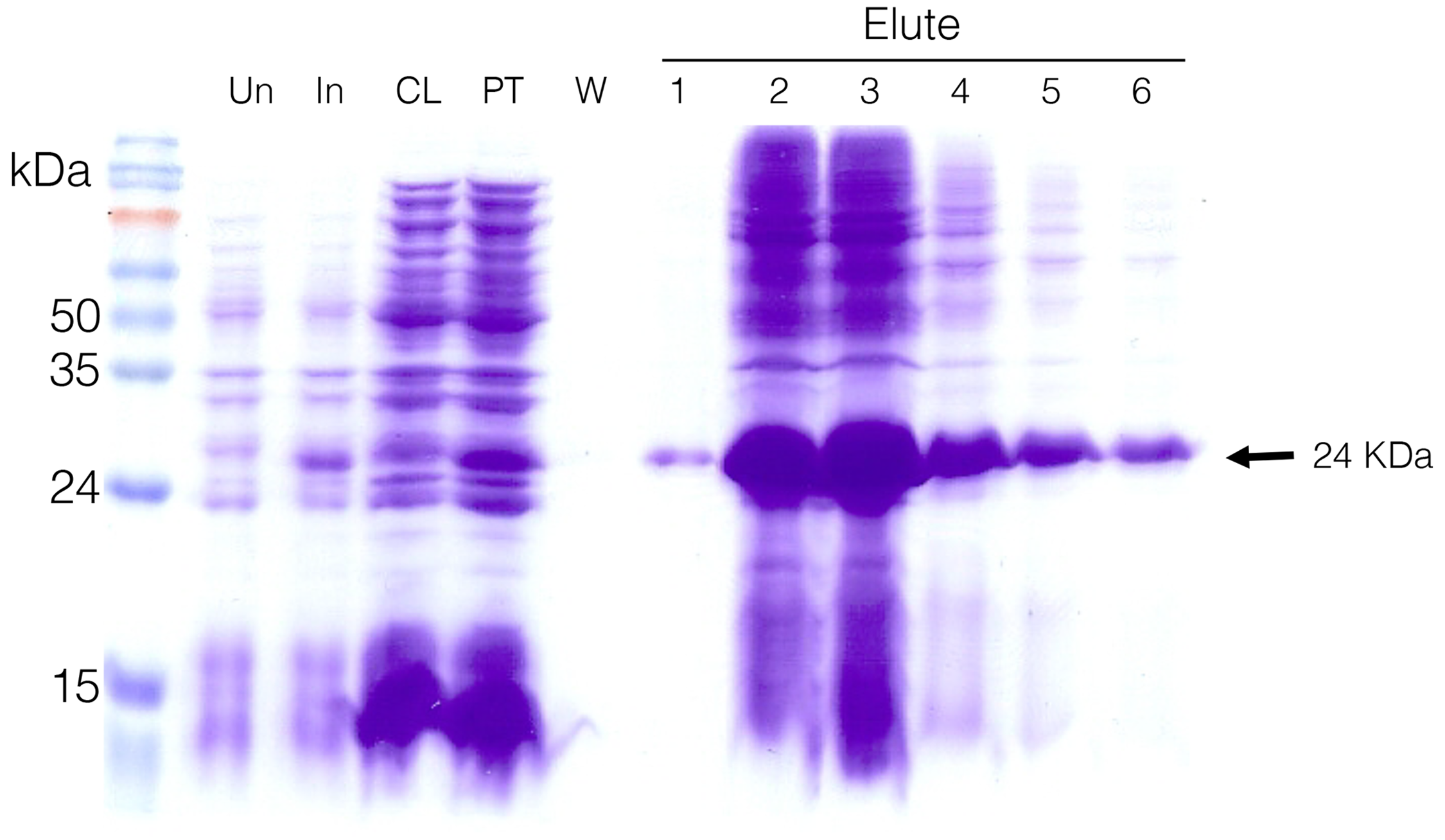

Supplement: S12 Fig — E. coli BL21[DE3]pLysS were transformed with plasmid DNA containing CsSBAT-chimeric cDNA. Recombinant CsSBAT-chimeric protein was induced by adding IPTG (isopropyl β-d-1-thiogalactopyranoside) into culture medium. The chimeric protein was purified using Ni-NTA column under denaturing condition. Un, uninduced total lysate; In, induced total lysate; CL, urea-treated clear supernatant; PT, pellet; W, the last washing; Eluate 1–6, the first to sixth fractions eluted from Ni-NTA column. (TIF) [file pntd.0008952.s012.tif]

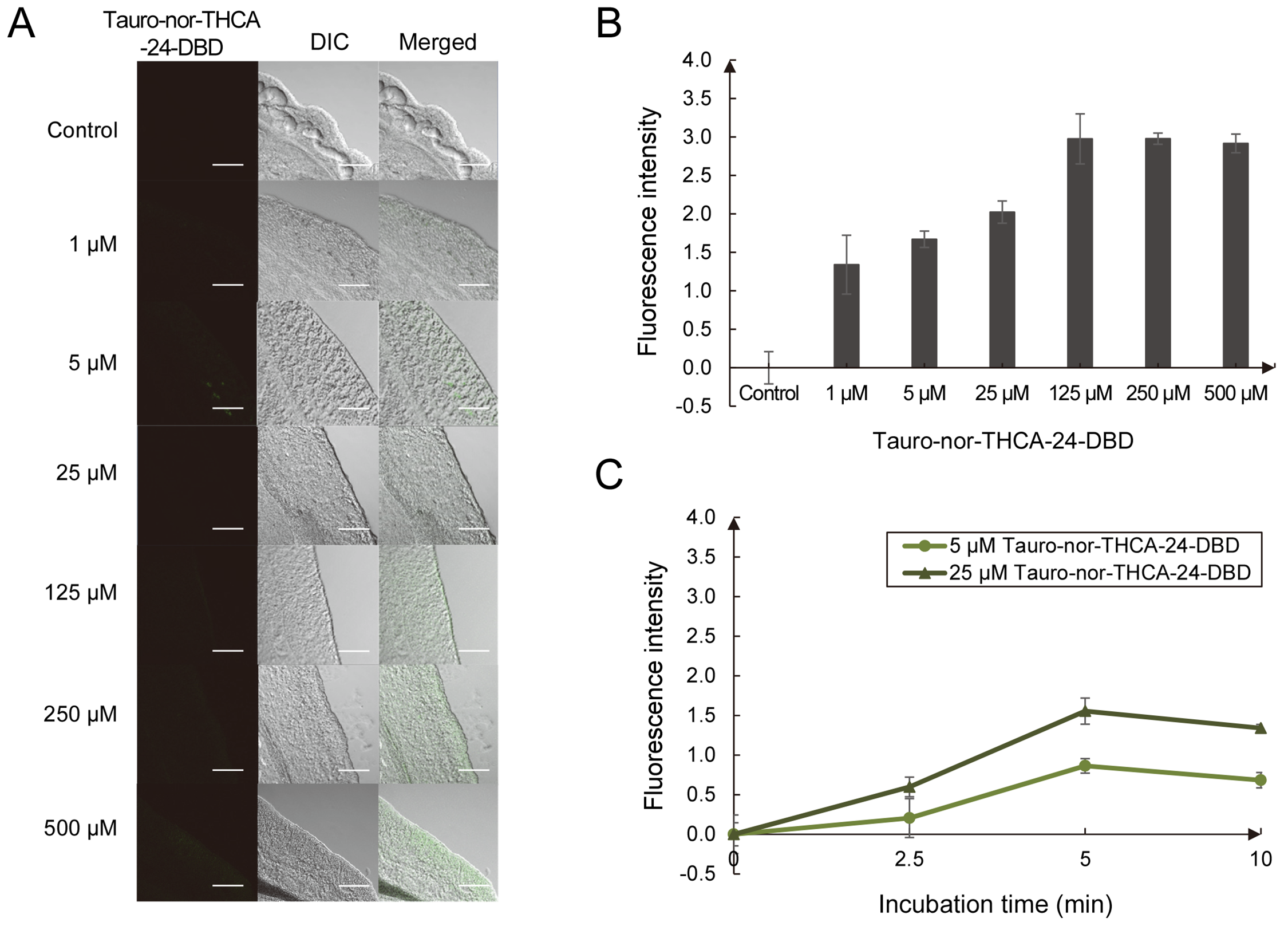

Supplement: S13 Fig — (A) Bile acid uptake in C. sinensis incubated in different tauro-nor-THCA-24-DBD concentrations. Scale bar = 80 μm. (B) Quantified fluorescence intensity based on panel (A). Fluorescence intensity of the negative control was subtracted from all groups. (C) Time course of bile acid permeation. No significant difference was observed between the two groups. (TIF) [file pntd.0008952.s013.tif]
